# *In vitro* discovery of promising anti-cancer drug combinations using iterative maximisation of a therapeutic index

**DOI:** 10.1038/srep14118

**Published:** 2015-09-22

**Authors:** M. Kashif, C. Andersson, S. Hassan, H. Karlsson, W. Senkowski, M. Fryknäs, P. Nygren, R. Larsson, M.G. Gustafsson

**Affiliations:** 1 Uppsala University, Dept of Medical Sciences, Cancer Pharmacology and Computational Medicine, Akademiska Sjukhuset, SE-751 85 Uppsala, Sweden; 2 Uppsala University, Dept of Immunology, Genetics and Pathology (Experimental and Clinical Oncology), Akademiska Sjukhuset, SE-751 85 Uppsala, Sweden

## Abstract

*In vitro*-based search for promising anti-cancer drug combinations may provide important leads to improved cancer therapies. Currently there are no integrated computational-experimental methods specifically designed to search for combinations, maximizing a predefined therapeutic index (TI) defined in terms of appropriate model systems. Here, such a pipeline is presented allowing the search for optimal combinations among an arbitrary number of drugs while also taking experimental variability into account. The TI optimized is the cytotoxicity difference (*in vitro*) between a target model and an adverse side effect model. Focusing on colorectal carcinoma (CRC), the pipeline provided several combinations that are effective in six different CRC models with limited cytotoxicity in normal cell models. Herein we describe the identification of the combination (Trichostatin A, Afungin, 17-AAG) and present results from subsequent characterisations, including efficacy in primary cultures of tumour cells from CRC patients. We hypothesize that its effect derives from potentiation of the proteotoxic action of 17-AAG by Trichostatin A and Afungin. The discovered drug combinations against CRC are significant findings themselves and also indicate that the proposed strategy has great potential for suggesting drug combination treatments suitable for other cancer types as well as for other complex diseases.

The development of a malignant tumour is generally associated with great complexity at the molecular level including the rewiring of different signal transduction pathways and biochemical feedback loops. This fact has made it widely accepted that in many cases a single drug treatment cannot produce the desired therapeutic effect^1^,^2^. Drug combinations are attractive owing to their lower propensity to trigger drug resistance (due to non-overlapping mechanisms of action) and side effects including adverse actions (due to lower dosage levels)^3^,^4^. Moreover, the use of combinations includes prospects of replacing some of the current, most expensive anti-cancer therapies by much cheaper drug cocktails. However, the huge space of possible compound mixtures is still largely unexplored and there is currently very slow progress in discovering novel, successful combination therapies for cancer.

A few combinations are indeed frequently used in the treatment of cancer, but such regimens are mostly based on empirical observations with agents already known to be effective in the disease rather than on well-defined and valid design criteria. The quite limited combination testing performed so far has covered a tiny fraction of all possible combinations and is therefore unlikely to result in selection of the optimal combinations among the huge number of possibilities^5^,^6^,^7^. Even in the current era of high throughput screening (HTS), the greatest challenge to achieve a successful, cost-effective search is the exponential growth in the possible number of drug and concentration combinations. Therefore, a procedure for successful discovery of multi-component therapies requires an semi-automated pipeline of the kind illustrated in Fig. 1A that involves integrated large-scale automated procedures and experimental readouts.

A few iterative computational-experimental procedures have recently been proposed that allow search in arbitrarily large combination spaces^8^,^9^,^10^. The general approach is illustrated in Fig. 1A. However, these seminal efforts do not provide any method designed explicitly to maximize a predefined therapeutic index (TI) that quantifies the desired effect versus the adverse side effects. In addition, the reported efforts are based on manual iterations rather than a semi-automated approach suitable for large scale experimentation.

In cancer pharmacology research, *in vitro* cancer cell line models are typically used with cytotoxicity or inhibition of proliferation as the primary read-out. For evaluation of drug combination effects, the current state-of-the-art uses a measure of synergy as endpoint because total cell kill is always trivially increased by adding another drug with cytotoxic action to a combination. Synergy as per classical definition is the improvement in efficacy over a theoretical model of expected joint action^11^. In the context of cancer, this stands in stark contrast to combination therapies used in clinical practice. Although there are some exceptions based on modern targeted drugs, still many anti-cancer combination therapies in use are not designed based on a synergistic interaction but from observed clinical utility in terms of increased survival. By using drugs with different side-effect spectrums, total dose can be increased while having limited side-effects. As already mentioned, the combination therapies are also attractive because they reduce the risk of emergent drug resistance. Neither of these two underpinnings of anti-cancer combination therapies are addressed by the use of classic measures of synergy. Thus a new principle for preclinical development of clinically relevant combination therapies could prove useful.

Here we report results from developing and employing a novel, semi-automatic, iterative search method that aims at finding optimal drug combinations for oncological targets that are maximising a clinically motivated TI. The TI serves as a proxy for the therapeutic benefit of a combination; an optimal combination should inhibit the cancer cells while least affecting healthy cells. The TI used is the differential cytotoxic action (in terms of cell viability) of a combination between cancer cell and normal/reference cell models^11^. We search for locally optimal drug combinations using an algorithm called MACS (Medicinal Algorithmic Combinatorial Screen)^9^, significantly improved in this work by taking experimental variability into account. We describe our pipeline in the context of applying it to CRC *in vitro* models.

Characterisation of five among the most promising drug combinations found by the pipeline suggests that all of them are suitable candidates for the treatment of CRC. One of these combinations, (Trichostatin A, Afungin, 17-AAG), was found to eradicate 6 different CRC model systems with limited side-activity against the normal/reference cells. It is also effective in primary cultures of tumour cells from CRC patients. Taken together, besides the discovery of a promising set of drug combinations for treatment of CRCs, this work provides one of the first successful semi-automated pipelines for discovery of anti-cancer drug combinations of arbitrary size with pronounced activity *in vitro*. As this method is fully generic, it enables and could inspire large-scale systematic explorations of the space of drug combinations for cancer in general, as well as for other complex diseases.

## Materials and Methods

All methods were performed in accordance with relevant guidelines and regulations.

### Liquid handling robot and programming environment

For liquid handling we used a Beckman Coulter Biomek 2000 liquid handling robot. We employed the integrated programming language BioScript Pro, a scripting language that is an extension of Tool Command Language (TCL)^12^. Using BioScript Pro, a “cherry picking” program was developed that takes an Excel file containing a list of desired combinations and their positions on the plate as input and performs the required combinatorial liquid handling. The program used and the associated parameter settings are available in the Supplementary Methods II (section on Beckman Coulter Biomek 2000 cherry picking program).

### Compounds

Thirteen drugs were used for the experiment using the MACS algorithm (first experiment, for details see Supplementary Table S1). These were selected on the basis of their diverse target of action in cancer cells and some of them (5FU, Oxaliplatin) are also clinically relevant. *IC*_20_ values (the concentration required to kill and/or inhibit growth of cells by 20% as compared to untreated control wells) of these drugs were estimated from concentration-response curves by using either curve fitting (in cases where the model could be properly fitted), or by manual inspection. Matlab code for estimation of *IC*_20_ values is provided in Supplementary Methods II (section on Matlab code for prediction of *IC*_10_ and *IC*_20_ values from concentration response data). In the main experiment, the following six drugs were used: Mitomycin, Sunitinib, Rapamycin, Trichostatin A, 17-AAG and Afungin. An *IC*_20_ value for each drug was calculated from concentration response curves in cell line HCT116 as above except for Afungin where the *IC*_20_ values were selected based on concentration-response data from the National Cancer Institute NCI-60 DTP Human Tumour Cell Line Screen (downloaded, Dec 2010: http://dtp.nci.nih.gov/docs/cancer/cancer_data.html). All drugs and their concentrations are shown in Table 1. The stock solution for each drug was prepared at a concentration 40 times higher than the *IC*_20_ values, so that desired *IC*_20_ concentration was restored in final volume inside wells of destination plates. Stock solutions were prepared in phosphate-buffered saline (PBS) and stored in 1 ml Eppendorf tubes (Eppendorf AG, Hamburg, Germany) at −70 °C until further use.

### Human cell lines

In the search using the MACS algorithm (first experiment), two human colorectal adenocarcinoma epithelial cell lines (DLD-1 and DLD-1KRAS/-) were used. These cell lines were obtained from Horizon Discovery Ltd, United Kingdom and cultured in McCoy’s 5A medium. In the main experiment using the TACS algorithm, the CRC cell lines HCT116 and HCT29 were obtained from American Type Culture Collection (ATCC, Rockville, MD) and were cultured in McCoy’s 5A medium. The leukemia cell line CCRF-CEM was a kind gift from WT Beck (Department of Pharmacology, college of Medicine, University of Tennessee, Memphis, TN). The cell line was grown in culture medium RPMI-1640 (HyClone, Northumbria, UK). SW620 was also obtained from ATCC and cultured in RPMI-1640 medium. RKO and HCT116KRAS/- cells were obtained from Horizon Discovery Ltd, United Kingdom and cultured in McCoy’s 5A medium. Normal (non-cancerous) colorectal cell line CCD 841 CoN was obtained from ATCC and cultured in EMEM medium. HCT116 cells labelled with GFP (green fluorescent protein) were obtained from Anticancer Inc (San Diego, CA, USA) and cultured in McCoy’s 5A medium.

### Tumour cell samples from patients

Tumour sampling of patients with solid tumours was performed intraoperatively during cancer surgery, whereas leukemia sampling was performed by vein puncture at routine blood sampling. All sampling was approved by the regional (Uppsala University) ethical committee (Dnr 2007/237) and after patient informed consent. Tumour cells from solid tumour tissue were prepared by collagenase digestion as previously described^13^. Leukemia cells were collected by Ficoll-Hypaque (Pharmacia, Uppsala, Sweden) gradient centrifugation as previously described^14^. The cells obtained from the leukemic samples were single cells or, for solid tumours, small cell clusters with ≥90% viability and with less than 30% non-malignant cells, as judged by morphological examinations of May-Grunwald-Giemsa-stained cytocentrifugate preparations.

### Spheroid-based experiments

Spheroids were formed using HCT116GFP cell line, as described previously^15^. Briefly, 10000 cells per well were seeded in 50 ml of fresh medium into 384-well F-bottom Corning Ultra-Low Attachment plates. Spheroids were cultured for 7 days without medium change prior to drug treatment. After treatment, cell viability was assessed by mean spheroid GFP fluorescence measurements, using ArrayScan VTI Reader (Cellomics Inc, Pittsburgh, PA, USA). The assay has been shown suitable for such measurements previously^15^,^16^.

### Calculation of SI values

The SI (Survival Index) values were calculated using the fluorometric microculture cytotoxic assay (FMCA)^17^,^18^ readout. FMCA measures the cell survival by measuring fluorescence. Fluorescence is produced by living cells with intact membranes by hydrolysis of fluorescein diacetate to fluorescein. Denoting the FMCA readout value by the letter *R*, the SI value is determined as the ratio (*R*_*treated*_ − *R*_*blank*_)/(*R*_*ctrl*_ − *R*_*blank*_) where *R*_*treated*_ denotes the readout for the well containing the treatment used, *R*_*blank*_ is the readout from a well without cells and *R*_*ctrl*_ is the readout from a well containing cells but no drugs.

### Statistical analyses

The 95% confidence intervals (CIs) for the mean were determined using the t-distribution. Assuming that the experimental variability is normally distributed, the difference between a pair of TI estimates has a t-distribution with the number of degrees of freedom being dependent on the number of replicates used to obtain the estimates, for details see Supplementary Methods Part III.

### mRNA gene expression analysis

Induced gene expression changes in the cell line HCT116 were analyzed using microarrays from Affymetrix ® after standard normalisation and pre-processing of data, for details see Supplementary Data, Gene Expression Data.

## Results

Initially, we applied the original MACS algorithm^9^ to find a drug combination that specifically targets cells that carry the clinically prevalent KRAS mutation in CRC. We used the difference in TI between CRC cell lines DLD-1 and DLD-1KRAS/- as a criterion to maximize. DLD-1 carries the clinically prevalent KRAS mutation whereas DLD-1KRAS/- has had the KRAS allele knocked out. The base set consisted of 13 compounds from different mechanistic classes (for details, see Supplementary Table S1) added at their *IC*_20_ concentrations. Generation 0 was initialised with a set of 14 randomly generated combinations (see Supplementary Tables S2). Cytotoxicity was measured by FMCA in both DLD-1 and DLD-1KRAS/- cell lines, and the *TI* of each combination was calculated as the difference *TI* = *S*_*KRAS*_ − *S*_*WT*_. Here *S*_*KRAS*_ and *S*_*WT*_ denote the SI values for DLD-1KRAS/- and DLD-1 (Wild type), respectively. A high value of TI corresponds to high cell kill in the KRAS mutation carrying cell line but low cell kill in the DLD-1KRAS/- cell line. The single best combination was then used to seed the next generation, using all one-compound perturbations around it. This was iterated until no gain in fitness could be made by such a local move. However, although the algorithm terminated with an optimal combination (see Supplementary Figure S1 and Supplementary Tables S2-S5), after examining this pilot run, it was considered necessary to take experimental variability into account as the TI gain observed between subsequent generations may simply be due to noise (experimental variability).

**Figure 1:**
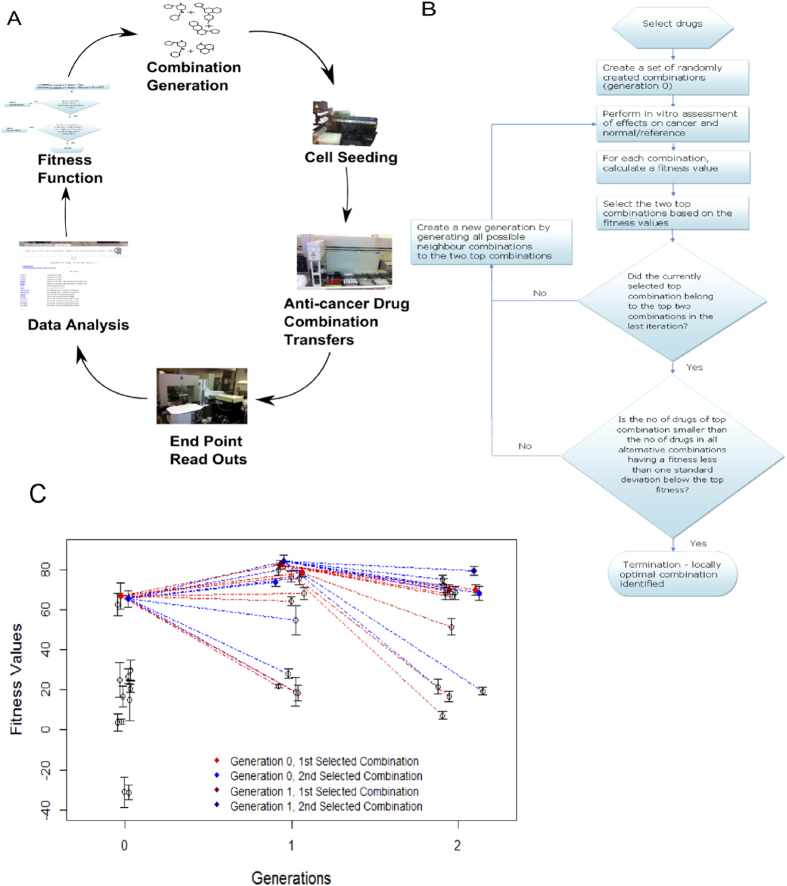
(**A**) Iterative search for drug combinations of arbitrary sizes. The procedure starts by generating an initial generation (population) of drug combinations randomly or guided by biological prior knowledge and assumptions. In each iteration the aim is to propose a new generation of drug combinations based on the results obtained so far. The procedure iterates through a number of generations until a stop criterion for a predefined fitness function is satisfied. (**B**) Overview of the TACS (Therapeutic Algorithmic Combinatorial Screen) algorithm designed and used in this work for iterative search towards promising drug combinations that offer a large TI value. The procedure iterates through a number of generations until a stop criterion is satisfied, taking experimental variability into account. (**C**) An example run of the TACS algorithm discussed in the main text. Generation 0 is initialised by a random selection of combinations. The drug combinations are assayed for cytotoxicity in three cell line models to determine a proxy for therapeutic benefit. The top two scoring combinations are selected to form the basis of the next generation. Red/blue lines indicate whether the combination was based on the first/second hit in the previous generation. The algorithm finds a set of improved combinations and terminates in two iterations. Notably, the top hit is actually derived from an ancestor that scored second best. One should also note that the two winners from generation 0 (blue and red) are present in all the three generations. Error bars indicate 95% confidence intervals.

Thus, we designed a less noise-sensitive procedure called Therapeutic Algorithmic Combinatorial Screen (TACS), see Fig. 1B, where we: (i) Take experimental variability into account in selecting seed combinations in each iteration (ii) Keep not only the best, but also the second best hit in each iteration as seeds to generate the next generation of drug combinations. (iii) Reduce experimental variability by performing the experiment twice for every generation and then determine mean TI values based on the pair of results obtained for each drug combination. (iv) Use a termination criterion that takes the experimental variability into account. (v) Encourage the selection of small combinations consisting of as few drugs as possible.

Instead of applying TACS to the same problem as in the pilot study, we decided to study perhaps an even more clinically interesting problem. Although identification of combinations that have activity exclusively in KRAS mutated cells (as aimed for in the pilot study) would be of outstanding interest, we think it would be even more interesting to find a combination which is designed to eradicate multiple types of CRC while, at the same time, inducing limited side-effects. Therefore, as described next, our main experiment was designed to find combinations with ability to eradicate two different CRC *in vitro* models while at the same time inducing limited side-effects in a reference/toxicity *in vitro* model.

### Implementation of novel TACS algorihtm

For our main experiment, now using our novel TACS algorithm (see Fig. 1B and Methods), we used two CRC cell lines, HCT116 and HT29 and modeled side-effects by the leukemia cell line CCRF-CEM. Using the same kind of notation as above, the TI used was


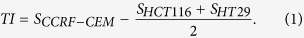


where *S*_*X*_ denotes the survival index of cell line X.

We hypothesize that the use of multiple (two) disease models in the fitness criterion increases the chance that the optimal drug combinations found will be widely applicable. More tentatively, the use of multiple cancer cell lines can also be regarded as a crude model of clone heterogeneity (although the different cell lines originate from different patients/genotypes). The six compounds used were 17-AAG, Afungin, Mitomycin, Sunitinib, Rapamycin and Trichostatin A, each having a different mechanism of activity (see Table 1, Methods). We initialised the search with 13 randomly selected combinations of the drugs at their *IC*_20_ concentrations and then iterated until the TI improvement was less than 1 standard deviation (SD). This search was biased towards small combinations by selecting the smallest combinations among those top-ranked but equal within experimental variability (again, using 1 SD as the criterion). The algorithm converged in two iterations, see Fig. 1C. Note that the optimal combination (Sunitinib, 17-AAG, Afungin, Trichostatin A) was produced by perturbing around the second best combination of generation 0 and would have been left out if only one combination would have been selected from generation 0, see Supplementary Tables S6-S8.

The resulting best combinations, (Sunitinib, 17-AAG, Afungin, Trichostatin A), yielded the fitness value 85 ±6.6 in generation 1, which is greater than the value 67 ± 4.9 obtained for the top hit of generation 0. This clearly suggests that the algorithm has succeeded in improving the TI criterion also when taking experimental variability into account. Furthermore, 5 combinations characterised in more detail were the two top winners from each generation (only five because one combination was the best in two consecutive generations), see Table 2.

### Generalisation of results to other concentrations and cell line models

**Figure 2:**
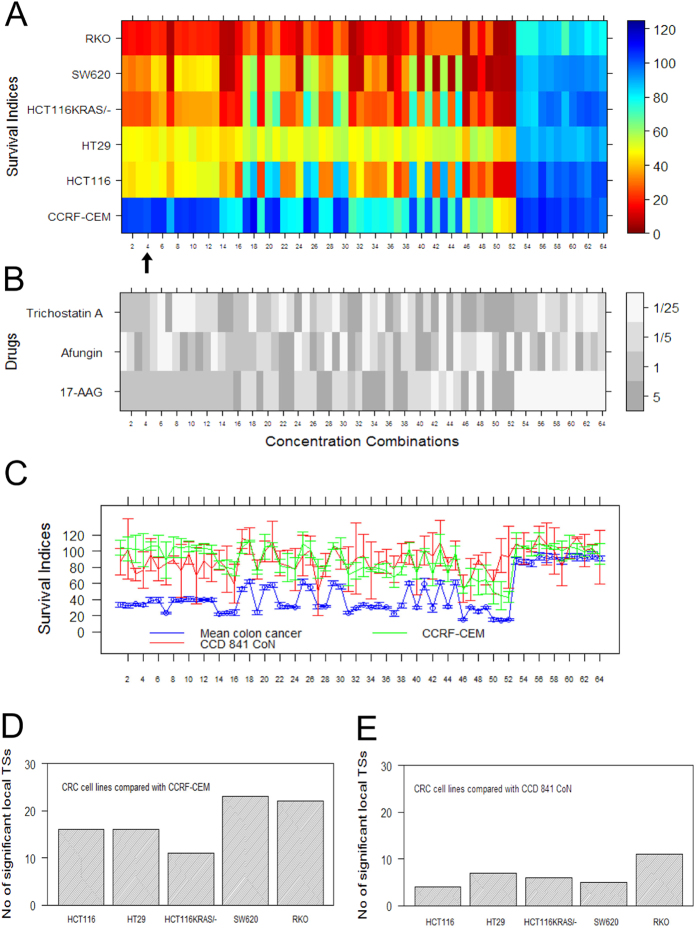
Factorial concentration-response study of combination (17-AAG, Afungin, Trichostatin A) and TS (therapeutic synergy). Each of 64 different concentration combinations was tested across five CRC cell line models and two normal/reference/toxicity cell line models. The concentrations are color coded in panel B and were selected to be 1/25, 1/5, 1 and 5 times the *IC*_20_ concentration used in the combination search. (**A**) Heatmap of SI values (%) for tested CRC cell lines, and the reference/toxicity model CCRF-CEM used in the iterative search, at the concentrations color coded in panel B. The arrow indicates location of the set of concentrations (1, 1, 1, 1) used in the search procedure using the TACS algorithm. (**B**) Heatmap of the 64 different concentrations tested, sorted by the fitness criterion in Eq (1) used by TACS. (**C**) Graph of average SI values (concentrations are shown in B) across the five cancer cell lines as well as SI values for the a normal (non-cancerous) colon cell line CCD 841 CoN and normal/reference/toxicity cell line model CCRF-CEM. Error bars indicate 95% confidence intervals. (**D**) Bar graph showing the number of significant local TSs detected when each CRC cell line is compared with CCRF-CEM. The largest possible number is 48 which would mean that there is a local TS for every concentration combination used. (**E**) Same kind of results as presented in Panel D but this time with the five CRC cell lines compared with normal (non-cancerous) colon cell line CCD 841 CoN.

One of the main challenges in anti-cancer drug discovery and cancer therapy is the heterogeneity between and within cancer tumours^19^. In order to determine to what extent the combinations discovered also are promising according to other CRC models, we performed a full factorial concentration-response study of the selected drug combinations using an extended cell line panel consisting of five CRC cell lines: HCT116, HT29, HCT116KRAS/-, SW620 and RKO. Using the combination (17-AAG, Afungin, Trichostatin A) as one illustrative example (for the other four combinations characterised, see Panels A, B & C of Supplementary Figures S2, S4, S6 & S8), each drug was tested at four different concentrations yielding in total 64 (4^3^) different concentration combinations as shown in Fig. 2B. The tested concentrations were 1/25, 1/5, 1 and 5 times the *IC*_20_ concentration used in the iterative search shown in Fig. 1C. The concentrations have been ordered by the fitness (TI) criterion in Eq (1) used by TACS. The heatmap in Fig. 2A shows SI values across the five CRC cell lines and the reference/toxicity model CCRF-CEM (rows) where each column corresponds to one concentration combination. It is evident that the combination effect generalises from the pair HCT116 and HT29 to the new three CRC cell lines HCT116KRAS/-, SW620 and RKO. In particular, it is clear that the drug combination has a stronger effect in the new cell lines HCT116KRAS/-, SW620 and RKO than in the original HCT116 and HT29 used in the iterative search. As the combinations are sorted according to TI, one also finds that the concentration combination used in the iterative search (indicated by the black arrow in panel A) is quite a good choice as only 3 other concentration combinations offered a (marginally) larger TI. However, increasing the concentration further decreased the TI by killing off the CCRF-CEM cells.

We also performed the corresponding full factorial concentration-response study using the normal (non-cancerous) colon cell line CCD 841 CoN and the leukaemia cell line CCRF-CEM. Figure 2C shows the average concentration-response across the five cancer cell lines panel together with SI values for these two cell lines. Clearly, many different concentrations for CCD 841 CoN and CCRF-CEM yielded positive TI values thus offering a promising therapeutic window (TW). It can be noted that TW provided by CCD 841 CoN overlaps with TW provided by CCRF-CEM.

### Therapeutic Synergy

An important indicator of the clinical utility of a drug combination is *in vitro* therapeutic synergy (TS). As already defined^11^, a drug combination offers TS if the largest TI it can achieve is larger than what can be obtained using any of its constituent drugs alone. For the drug combination (17-AAG, Afungin, Trichostatin A) we made all 10 possible pairwise comparisons of the effect of the combination in one of the five CRC cell lines with the corresponding effect in one of the two normal/reference/toxicity cell lines. For each of these 10 pairwise drug comparisons the number of concentration combinations found to yield a TI significantly larger than the largest TI produced by any of single drugs was determined (using t-test and a Bonferroni corrected p-value threshold), see Panels D and E of Fig. 2. Each bar indicates the total number of concentration combinations that yielded a significantly higher TI for the drug combination than for any single constituent drug within the concentration range studied. The largest possible number of such local TSs achievable is 48 which would have occurred if every pair tested resulted in a significant local TS. For each bar (pairwise comparison), a p-value was calculated and found to be less than or equal to 10^−4^. These p-values are associated with a previously developed omnibus test^11^ specifically designed to avoid the multiple-testing problem via the null hypothesis that there is no TS (thus assuming there is no drug concentration combination that obtains a higher TI than any of the constituent drugs alone). For the other four combinations characterised for TS, see Panels D & E of Supplementary Figures S2 & S8 and Panel D of Supplementary Figures S4 & S6.

### Efficacy in primary cultures of tumour cells from CRC patients and in a spheroid microtumour *in vitro* model

**Figure 3:**
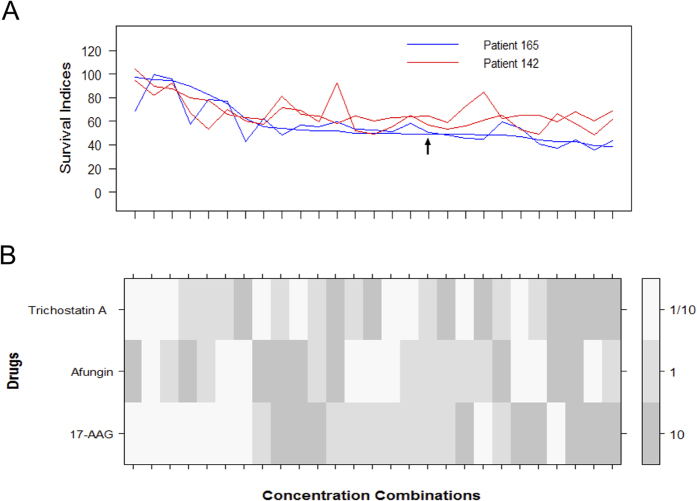
Factorial concentration-response study of combination (17-AAG, Afungin, Trichostatin A) in patient cells. This study was performed at three different concentrations corresponding to 1/10, 1 and 10 times the concentration used in the iterative search. (**A**) SI values for CRC patient cells as obtained in two experiments per patient sample. The black arrow shows the concentration combination discovered during the original iterative search. (**B**) Concentrations corresponding to A expressed as fractions of the concentrations used the iterative search. The concentration combinations were sorted according to the first run performed for patient 165.

The efficacy of the combination (17-AAG, Afungin, Trichostatin A) was investigated in primary cultures of tumour cells from metastases of n = 11 patients with CRC. For comparison, these data were compared with those of tumour cells from patients with ovarian (n = 9) or kidney (n = 6) cancer as well as in one patient with lymphoma. Cytotoxicity induced by the combination in 2 of the CRC samples is shown in Fig. 3, for results regarding the other four combinations see Supplementary Figures S3, S5, S7 & S9. Due to the limited number of cells available for analysis, for these two samples only three different concentrations were used corresponding to 1/10, 1 and 10 times the concentrations used in the iterative search. A summary of the cytotoxicity induced by the combination in all the samples (ovarian, kidney and CRC) is shown in Supplementary Figure S10. Overall, the CRC samples were more resistant than ovarian and lymphoma samples and essentially as resistant as the kidney cancer samples. This pattern of sensitivity *ex vivo* in tumour cell samples from patients corresponds well to that of standard cytotoxic drugs used in the clinic^20^. The activity of the combination differed considerably between the individual CRC samples. This is also in line with the varying sensitivity to standard drugs in patient samples, reflecting the biological heterogeneity between tumour samples from the same tumour types. The difference in sensitivity among the CRC samples was not obviously associated with patient treatment status. Thus, there were both sensitive and resistant samples from treatment naïve and previously treated patients (not shown).

Interestingly, the combination was active at relatively low concentrations of the drug components compared to the concentrations of standard cytotoxic drugs needed to induce significant cytotoxicity in patient tumour cells during identical assay conditions. In the combinations all the three constituent drugs were used at concentrations that correspond to levels below *IC*_20_ when used in HCT116 cells. Since primary patient cells generally are more resistant than cell lines, this means that the constituent drugs would have a very limited effect on the patient cells (killing far less than 20% of the cells) if they were to be used individually. Two examples illustrating that patient cells are more resistant can, for example, be found in our previous work^11^,^20^. In Fig. 3 of the work by Kashif *et al.*^11^, one finds that the *IC*_20_ values of the standard drugs 5-FU and Oxaliplatin in HCT116 cells are approximately 30 and 16 μM, respectively, whereas in Fig. 1 of the article by Cashin *et al.*^20^ one finds that the corresponding concentrations in primary patient cells are approximately 500 and 30 μM.

**Figure 4:**
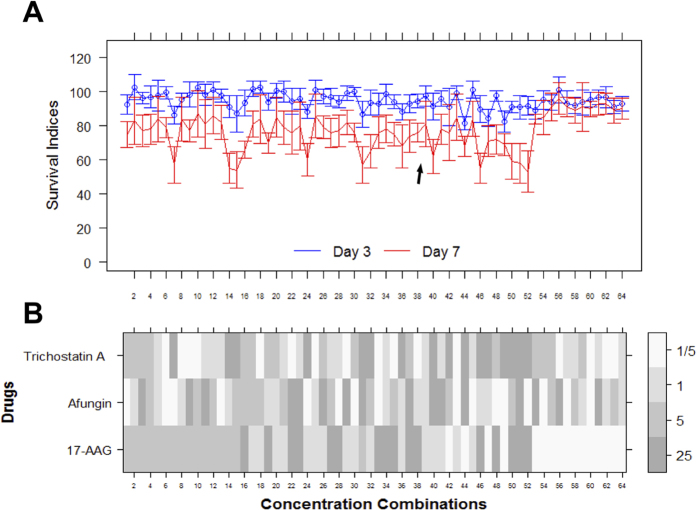
Factorial concentration-response study of combination (17-AAG, Afungin, Trichostatin A) in a HCT116 spheroidal model. (**A**) Profiles of SI values (%) for the spheroid model at multiple time points. The SI values are sorted in the same order as in Fig. 2 (although all concentrations here are 5 times higher). The black arrow indicates the concentration combination discovered during the original iterative search. (**B**) Concentrations corresponding to A expressed as fractions of the concentrations used in the iterative search.

Finally, we also performed a concentration-response characterisation of the combination in a GFP-labelled HCT116 spheroidal model (Fig. 4A). Three-dimensional spheroids of cells from the cell line HCT116GFP were created in-house and shown to be generally more resistant to standard cytotoxic drugs than monolayer (2D) cultures^15^. The spheroids were treated according to a full factorial concentration-response experiment at concentrations 5 times higher than for the 2D models and the resulting SI values were quantified every 24h using measurement of mean spheroid GFP fluorescence. In Fig. 4A, the temporal (3 & 7 days) evolution of the SI values for the combination (17-AAG, Afungin, Trichostatin A) are displayed, showing that it also has effect in spheroidal models in the concentration range studied.

### Systemic mRNA gene expression analysis

We performed systemic micoarray mRNA gene expression analysis of the changes induced by the combination (17-AAG, Afungin, Trichostatin A) in HCT116 cells to gain more insights about its mechanism of action. Induced changes in mRNA signatures for the combination as well as its constituent drugs were derived from the fold change in expression relative to vehicle control in HCT116 cells while following the Connectivity Map (CMap) protocol^21^. Table 3 shows the top ten most similar compounds in terms of mRNA expression signature produced by a search against the publicly available CMap database. The pattern clearly suggests the mode of action is proteotoxic, specifically inhibition of HSP90, with all top 5 hits having been described as HSP90 inhibitors^22^,^23^,^24^,^25^,^26^. Since 17-AAG (denoted as Tanespimycin in CMap) is part of the combination and known to be a HSP90 inhibitor and since HSP90 inhibitors are enriched in analysis, we propose that the main mechanism of actions is protetoxicity mediated through HSP90-inhibition. In this context one should also note that Trichostatin A is an indirect HSP90 inhibitor because its inhibition of HDAC6^27^ leads to HSP90 deactivation^28^. Inhibition of HDAC6 also means that cells fail to clear misfolded protein aggregates from the cytoplasm, cannot form aggresomes properly, and are hypersensitive to the accumulation of misfolded proteins^28^,^29^.

For more details regarding the gene expression analysis, see Supplementary Methods Part I. Using the mRNA gene expression signatures obtained, additional bioinformatics searches were performed using commercial tools like MetaCore^*TM*^ (www.thomsonreuters.com) and Nextbio (www.nextbio.com) as well as open access alternatives like Gene set enrichment analysis (www.broadinstitute.org), gene ontology (www.geneontology.org), and g:Profiler^30^,^31^. However, no additional outstanding conclusions were made.

The closest match is Withaferin A (WA) which recently was reported by Koduru *et al.* to inhibit cell viability in three CRC cell lines (HCT116, SW480, and SW620) in a dose-dependent manner while at the same time not having any significant effect on normal colon epithelial cells (FHC)^32^. Thus WA and the combination (17-AAG, Afungin, Trichostatin A) show strikingly similar phenotypic effects in the context of CRC cell lines and normal colon cell lines. However, instead of HSP90-inhibition, Koduru *et al.* suggest and offer experimental results supporting that WA inhibits Notch-mediated prosurvival signaling that facilitates c-Jun-NH2-kinase mediated apoptosis in colon cancer cell lines.

As discussed in the review by Vanden Berghe *et al.* the heat shock regulating activity of WA is still very unclear as WA has been reported to inhibit heat shock protein activity and also found to be a highly potent heat shock response inducer in screen covering 80000 natural and synthetic compounds. The authors suggest that this might be a consequence of concentration of WA applied as well as the cellular context. For example, the induction of heat shock response by WA might be secondary to WA-mediated HSP90-inhibition. WA is an unusually difficult compound to grasp as it has been reported to exert anti-inflammatory, pro-apoptotic, pro-autophagic as well as anti-invasive and anti-angiogenic effects. It has also been shown to have a remarkably wide range of molecular/cellular targets including vimentin, Bcl-2, the proteasome, NF*κ*B, Akt, Stat3, kinase activity, notch signalling, endoplasmic recticulum stress, reactive oxygen species (ROS) production and heat shock response activity^33^,^34^. Of particular interest in this context is the reported HSP90 inhibitor activity of WA in pancreatic carcinoma cells^22^ and breast cancer cells^35^ and improved mechanistic understanding regarding this effect^33^,^36^.

## Discussion

As indicated in the introduction, the space of drug combinations beyond pairs of drugs has outstanding potential but is almost completely unexplored using systematic approaches. Since the wet laboratory work grows exponentially when exploring this space systematically, semi-automation seems to be the only feasible way forward. There are many multi-disciplinary challenges and pitfalls towards obtaining successful use of semi-automated procedures that are able to speed up the search for clinically promising drug combinations of arbitrary size. Taken together, the contributions presented here not only demonstrate the potential of this semi-automated approach but also provide a set of promising combinations that deserve further characterisation as candidates for cancer treatment, notably CRC. More specifically, the main contributions may be briefly summarised as follows: (1) Development and deployment of a novel semi-automated and successful computational-experimental pipeline for combination studies. (2) An independent validation of one of the first among very few search methods reported for drug combinations of arbitrary size (MACS) and clinically and practically important improvements of this approach. (3) The proposed use of two innovative *in vitro* model systems suitable for drug as well as drug combination screening for positive TI (pairs of cell lines having the same genetic background and multiple cell line models of cancer and normal/reference/healthy patient samples). (4) Discovery and characterisation of several drug combinations for CRC treatment showing promising activity in an extended panel of cell line models, in a 3D spheroidal model and in primary cultures of patient CRC cells.

There are multiple reasons for combination therapies. As also mentioned in the introduction, combination therapies are receiving increased attention and there are multiple reasons for this. Often the goal is to find a combination that displays synergy in the sense that the combination shows greater effect than expected from the effects of the constituent drugs. Economically, combinations are promising in the context of drug repurposing as a combination of approved drugs may target diseases for which the constituent drugs are not registered or may offer a much cheaper alternative than those currently used in the clinic. The time for a combination consisting of already FDA (Food and Drug Administration) approved drugs to reach the market is much shorter than for a novel drug candidate, since the constituent drugs have already passed clinical trials. Another commonly cited motivation for combinations is the complexity of the interacting network causing many diseases, prominently cancer. That is, combinations seem or are claimed to be needed to achieve therapeutic effects, since a single targeted drug is insufficient to target diseases driven by complex networks.

This work presents a generic framework for discovering anti-cancer drug combinations. Except for clear cut examples such as when an individually inactive drug increases the effect of the combination, synergy is a non-trivial concept to define. In addition, classical definitions of drug synergy do not correspond to clinical reality as clinically used combination therapies often display limited or no synergistic effects when investigated using *in vitro* models. We postulate that the clinical value of combination therapies for cancer may derive from being able to increase the total drug exposure and/or from the potency to rewire complex networks while maintaining side-effects at an acceptable level. Systematic search for promising combination therapies has conventionally been limited to consider combinations of only two drugs, where typically a potential synergistic partner for an investigational drug is evaluated. Here we have presented a framework that allows searching for combinations of a potentially arbitrary number of drugs.

In connection to the results presented, there are some algorithmic issues to consider. The TACS algorithm as presented is limited by the number of one-off (one drug away) neighbouring combinations to evaluate in each generation. However, we do not think the exact details of the optimisation algorithm are crucial. Also, we used fixed concentrations when searching for combinations. What we do think is crucial though is to take experimental variability into account as to avoid jumping around randomly among a set of combinations equally potent according to the estimated performance. Moreover, although the search space in our main example consisted of just 57 = 2^6^ − 7 possible combinations of 2 or more drugs, our semi-automatic pipeline allows for much larger search spaces. The main limitation on the size of the library used is the ability to exhaustively evaluate all nearest (one modification away) combination neighbours in the iteration. Therefore, in practice the TACS algorithm seems most suitable for finding combination therapies from relatively small (<50) sets of compounds. For larger sets, alternative, less-exhaustive search strategies are required, which is a topic for future work.

The selection of a drug set for the TACS algorithm can be made in a variety of ways. For exploratory (*de novo*) studies and when there is no obvious prior knowledge to exploit, drugs can be selected more or less randomly without any prior hypothesis about their mechanism of action. However, the selection can also be based on a hypothetical model of the pathology in combination with detailed knowledge about the targets/mechanisms of particular drugs. For example one may select a set of drugs for specific targeting of one particular signal transduction pathway or biological process. In summary, since TACS is a generic combination search algorithm, the drug set used can be tailored to the particular needs of the study of interest.

The current implementation of TACS uses fixed doses of the selected set of drugs. However, it is possible to use the same drug at multiple concentrations using TACS simply by interpreting each concentration as a different drug in the TACS implementation. Additionally, if a combination is producing a large value of TI at a fixed concentration this does not directly ensure the presence of a TW as well. Therefore, one should perform validation studies to find out the existence of large TWs.

A few aspects of the clinical relevance of the presented approach and results are interesting to note. When characterizing the concentration-response surface of the combinations discovered, we find positive TI values for a range of concentrations making administration promising. Clinical use would be nearly impossible if the constituent drugs all have narrow TWs. Of course, the choice of normal (or reference/toxicity) cell models used in the iterative search to a large extent determines the possibilities to find clinically relevant combinations. The use of CCRF-CEM, a T-cell lymphoblast cell line established from an acute lymphocytic leukemia as a normal/reference/toxicity cell model is a potential weakness. However it does show generally higher chemosensitivity compared to cell lines derived from solid tumours like CRC. Sensitivity of the CCRF-CEM can also be verified from the National Cancer Institute NCI-60 DTP human tumour cell line screen database. More generally, hematologic toxicity is well known to be one of the main dose-limiting factors in clinical practice. Therefore, it may be viewed as a general marker of non-specific cytoxicity that should be avoided. We believe this interpretation and use of CCRF-CEM is strengthened by us subsequently finding limited therapeutic effects also in the normal (non-cancerous) cell line CCD 841 CoN. Lastly, it is encouraging to conclude that the combinations offer therapeutic synergy, i.e., the therapeutic benefit cannot be achieved using any of the constituent drugs on their own. Applicability of the overall iterative search strategy is further strengthened by finding a combination to have activity in CRC patient primary cell cultures within the range of, comparatively very low, concentrations explored.

The combination was also tested in tumour cell samples from patients with ovarian cancer (n = 9), kidney cancer (n = 6) and lymphoma (n = 1). From the clinic these tumour types are expected to show considerable differences in drug sensitivity, with lymphoma being sensitive, ovarian cancer intermediate and renal cancer very resistant. Interestingly, the activity of the combination was essentially equal in these diagnoses, indicating it is targeting a general mechanism in cancer cells (data not shown). Furthermore, the combination was active at relatively low concentrations of each component compared with standard drugs, and without much concentration dependence within the combination. This indicates high potency of the combination with early saturation of the target mechanism. However, it is not possible on the basis of our relatively few observations to draw any strong conclusions regarding the activity of the combination in tumour samples from patients. Finally, the clinical potential of the combination is also supported by preliminary results suggesting that the combination induces cytotoxicity in a spheroid model.

One of the potential weaknesses of the current study is that *in vitro* measurements have limited clinical relevance^37^,^38^. Therefore, how combinations will behave in patients (clinical trials) is a question for further studies. Obviously the current study would also benefit from the use of additional *in vitro* non-cancer and cancer models both during the search phase and the follow up characterisation of the most promising combinations found (where results from *in vivo* models also would be of great interest). However this is beyond the scope of the present paper being focused on demonstrating the general potential of the approach suggested (i.e. to take experimental variability into account when employing the semi-automated search pipeline developed). Moreover, regarding the suggested hill climbing algorithm introduced (TACS) there are obvious possibilities for further algorithmic improvements that may result in even more robust and/or faster search. For example, in future studies it would be interesting to compare this type of local search methods with global optimisation methods including genetic algorithms. Moreover, it could prove beneficial to explore the possibilities to enhance the search performance by integrating prior knowledge about the drug combination search space as well as about the experimental variability associated with the experiments performed. Furthermore, in this context we would also like to make a reminder that the whole idea of searching for promising drug combinations beyond pairs of drugs often is considered as a weakness (for various reasons including potential difficulties reaching the clinic). However, as already discussed above, the potential of drug combinations beyond pairs of drugs is largely unexplored and deserves further preclinical evaluation. In this effort, new algorithms of the kind suggested and explored here will be required.

The mechanism of action of our example combination (17-AAG, Afungin, Trichostatin A) is clearly suggested to be HSP90-inhibition. It seems as if Trichostatin A and Afungin potentiate the effect, but especially the role of Afungin is unclear. We performed additional analyses of the combination induced mRNA expression changes, sifting out the set of probes perturbed uniquely by the combination. However, using various bioinformatics tools for pathway analysis (see above) no outstanding pathway or function could be discerned among the annotations. One should also note that the closest compound matching when performing a CMap based systemic mRNA analysis was Withaferin A (WA) which recently was reported by Koduru *et al.* to inhibit cell viability in three CRC cell lines (HCT116, SW480, and SW620) while at the same time not having any significant effect on normal colon epithelial cells (FHC)^32^. The same report also suggests that WA achieves its effect via inhibition of Notch-mediated prosurvival signaling, thus not via HSP90-inhibition. Further investigations into the mechanism of the combination are ongoing.

## Additional Information

**How to cite this article**: Kashif, M. *et al.* *In vitro* discovery of promising anti-cancer drug combinations using iterative maximisation of a therapeutic index. *Sci. Rep.* **5**, 14118; doi: 10.1038/srep14118 (2015).

## Supplementary Material

Supplementary Information

Supplementary Methods

Supplementary Data

Supplementary Gene expression data

Supplementary Movie S1

Supplementary Movie S2

Supplementary Movie S3

## Figures and Tables

**Table 1:** Drugs used in the iterative search using the TACS algorithm.

Drug	Suggested Activity	Obtained from	Stock Conc.	Final Conc.
Sunitinib	Tyrosine kinase inhibitor^39^	Lc Laboratories USA	126.5 μM	3.16 μM
Rapamycin	mTOR (mammalian target of rapamycin) inhibitor^40^	Sigma-Aldrich Sweden AB	86.4 μM	2.16 μM
Trichostatin A	Histone deacetylase inhibitor^41^	Sigma-Aldrich Sweden AB	3.2 μM	0.08 μM
17-AAG	Heat shock protein (HSP) inhibitor^42^	Lc Laboratories USA	40 μM	1 μM
Mitomycin	Antitumour antibiotics^43^	Sigma-Aldrich Sweden AB	14.9 μM	0.37 μM
Afungin	Antifungal substance	Sigma-Aldrich Sweden AB	0.4 μM	0.01 μM

Stock solution for all drugs were prepared 40 times higher than the final concentrations that were restored when diluted with cell suspension in the wells of the experimental plate.

**Table 2:** Characterisation of the two top combinations obtained in each of the three generations.

Sr No	Combination	TI	Comments	TS CCD 841 CoN	TS CCRF-CEM
1	Sunitinib, 17-AAG, Afungin, Trichostatin A	85±6.6	Generation 1&2, best combination	5	3
2	Rapamycin, 17-AAG, Trichostatin A	83±4.9	Generation 1, 2nd best combination	–	2
3	17-AAG, Afungin, Trichostatin A	75±5.4	Generation 2, 2nd best combination	5	5
4	Rapamycin, 17-AAG	67±4.9	Generation 0, best combination	–	2
5	Sunitinib, 17-AAG, Afungin	65±8.8	Generation 0, 2nd best combination	5	0

Only five different combinations were characterised since the combination Sunitinib, 17-AAG , Afungin, Trichostatin A was the best in two consecutive generations. These five combinations with their corresponding TIs obtained in the respective generations (where they were among the top two winners) are shown as 95% confidence intervals. In the last two columns results of subsequent follow-up experiments using five CRC cell lines and two different normal/reference cell lines are summarised. Each column shows the number of times there is TS (therapeutic synergy) when comparing pairs of one CRC cell line and the corresponding normal/reference cell line which is either CCD 841 CoN (normal colon) or CCRF-CEM (leukemia). Since 5 different CRC cell lines were used during the follow-up experiments, no more than 5 comparisons can exist that have significant TS. When there is no data available the missing result is indicated as –. For example, one may find that the third combination is producing the maximum number of significant TS.

**Table 3:** Best matching compounds in Connectivity Map (CMap) when using the mRNA gene expression signature of the combination (17-AAG, Afungin, Trichostatin A) as a query.

Rank	Compound name and no of instances	mean score	p-value
1	Withaferin A (n = 4)	+0.70	<10^−5^
2	Geldanamycin (n = 15)	+0.58	<10^−5^
3	Alvespimycin (n = 12)	+0.56	<10^−5^
4	Tanespimycin (n = 62)	+0.49	<10^−5^
5	Monorden (n = 22)	+0.43	<10^−5^
6	Alcuronium chloride (n = 2)	−0.82	6·10^−4^
7	Emetine (n = 4)	−0.62	8·10^−4^
8	Flunixin (n = 5)	−0.59	8·10^−4^
9	Disulfiram (n = 5)	+0.62	10^−3^
10	Diethylstilbestrol (n = 6)	+0.54	10^−3^

The mean connection score shown is produced by the publicly available CMap signature matching algorithm. The ranking is first based on p-values provided and then on the mean score value which is based on n instances of the compound in the database.
