# Supplementary material for: In vitro discovery of promising anti-cancer drug combinations using iterative maximisation of a therapeutic index
Source: Sci Rep. 2015 Sep 22;5:14118. doi: 10.1038/srep14118 (PMC4585751; doi:10.1038/srep14118)
Supplement: Supplementary Information [file srep14118-s1.pdf]

OVERVIEW OF THE SUPPLEMENTARY MATERIAL OF THE ARTICLE

In vitro discovery of promising anti-cancer drug combinations using maximization of a therapeutic index

M. Kashif[1], C. Andersson[1], S. Hassan[1], H. Karlsson[1], W. Senkowski[1], M. Fryknäs[1], P. Nygren[1], R. Larsson[1], M.G. Gustafsson[1,*]

[1]Uppsala University, Dept. Medical Sciences Cancer Pharmacology and Computational Medicine, Academic Hospital, 751 85 Uppsala, Sweden

[2]Uppsala University, Dept of Radiology, Oncology and Radiation Science, Academic Hospital, SE-751 85 Uppsala, Sweden.

*Corresponding author: Mats.Gustafsson@medsci.uu.se

The supplementary material is divided into 4 files and 3 movies, these are 1-SupplementaryData.pdf, that includes

a) Table S1: A set of 13 clinical/experimental drugs used in the iterative search using the MACS algorithm (pilot experiment).
b) Table S2: Results of generation 0 of pilot experiment.
c) Table S3: Results of generation 1 of pilot experiment.
d) Table S4: Results of generation 2 of pilot experiment.
e) Table S5: Results of generation 3 of pilot experiment.
f) Table S6: Results of generation 0 of main experiment.
g) Table S7: Results of generation 1 of main experiment.
h) Table S8: Results of generation 2 of main experiment.
i) Figure S1: Optimization overview of all iterations of pilot experiment.
j) Figure S2: Factorial concentration-response and TS (therapeutic synergy) study of combination (Sunitinib, 17-AAG, Afungin, Trichostatin A).
k) Figure S3: Factorial concentration-response study of combination (Sunitinib, 17-AAG, Afungin, Trichostatin A) in patient cells.
l) Figure S4: Factorial concentration-response and TS study of combination (Rapamycin, 17-AAG, Trichostatin A).
m) Figure S5: Factorial concentration-response study of combination (Rapamycin, 17-AAG, Trichostatin A) in patient cells.
n) Figure S6: Factorial concentration-response and TS study of combination (Rapamycin, 17-AAG).
o) Figure S7: Factorial concentration-response study of combination (Rapamycin, 17-AAG) in patient cells.
p) Figure S8: Factorial concentration-response and TS study of combination (Sunitinib, 17-AAG, Afungin).
q) Figure S9: Factorial concentration-response study of combination (Sunitinib, 17-AAG, Afungin) in patient cells.
r) Figure S10: Analysis of patient data for the combination (17-AAG, Afungin, Trichostatin A).

2- SupplementaryMethods-I-II-III.pdf, consists of
Supplementary Methods I

a) Gene expression analysis of combination (17-AAG, Afungin, Trichostatin A).
b) Statistical variability calculations.
c) Performance monitoring of experimental/computational pipeline.
d) Supplementary Figure S11: Performance monitoring of experimental/computational pipeline.

Supplementary Methods II

a) Beckman Coulter® Biomek 2000 cherry picking program
b) Matlab® code for prediction of IC10 and IC20 values from concentration response data.
c) Supplementary Figure S12: Cell plate layout.

Supplementary Methods III

a) Theory for determination for the number of degrees of freedom related to t-test for therapeutic synergy.

3- Gene Expression Data.xls, includes

a) Contains differential gene expression signature of combination (17-AAG, Afungin, Trichostatin A).
b) Compounds matching that signature when run CMap 02 (https://www.broadinstitute.org/cmap/).

4- Movies

a) Supplementary Movie S1.mov, combination (17-AAG, Afungin, Trichostatin A) treatment of $MelJuso^{YFP}$.
b) Supplementary Movie S2.mov, $MelJuso^{YFP}$ treated with 17-AAG.
c) Supplementary Movie S3.mov, $MelJuso^{YFP}$ treated with Bortezomib.
