# Supplementary material for: In vitro discovery of promising anti-cancer drug combinations using iterative maximisation of a therapeutic index
Source: Sci Rep. 2015 Sep 22;5:14118. doi: 10.1038/srep14118 (PMC4585751; doi:10.1038/srep14118)
Supplement: Supplementary Methods [file srep14118-s2.pdf]

Supplementary Methods Part I: In vitro discovery of promising anti-cancer drug combinations using maximization of a therapeutic index

M. Kashif[1], C. Andersson[1], S. Hassan[1], H. Karlsson[1], W. Senkowski[1], M. Fryknäs, [1]. Nygren[2], R. Larsson[1], M.G. Gustafsson[1,*]
Uppsala University, Dept Medical Sciences Cancer Pharmacology and Computational Medicine, Academic Hospital, 751 85 Uppsala, Sweden
*Corresponding author: Mats.Gustafsson@medsci.uu.se

August 13, 2015

1 Brief Description

This supplement includes gene expression analysis of combination (17-AAG, Afungin, Trichostatin A), statistical variability calculations and performance monitoring of experimental/computational pipeline.

2 Gene expression analysis

2.1 Extraction of combination (17-AAG, Afungin, Trichostatin A) differential gene expression

Gene expression analysis of CRC cell line HCT116 treated with combination (17-AAG, Afungin, Trichostatin A) for 6 hours and untreated control was made by using Affymetrix GeneChip® expression arrays (Human Genome U 133 Plus 2.0). Raw data was normalized in the free software Expression Console provided by Affymetrix (http://www.affymetrix.com) using the MAS 5.0 algorithm. Only those probe set IDs were selected in combination differential gene expression signature (see supplementary Material Gene Expression Data) where there was two fold changes in the ratio of treated vs control (abs[log2(treated/control)] $>$ 1).

2.2 Connectivity Map

Connectivity Map version 2 (CMap02), (https://www.broadinstitute.org/cmap/) is a database of more than 7,000 expression profiles resulted with treatment of 1309 compounds. Combination differential expression was searched in CMap02 for similarities. Detailed results are available in the Supplementary Material Gene Expression Data where it can be seen that most of the top ranked compounds like monorden, alvespimycin, tanespimycin and geldanamycin are proteotoxic and HSP90 inhibitors.

2.3 MetaCore™

Analyses using MetaCore™ support the CMap02 based findings that the combination initiates a protein folding response to unfolded proteins (p=1.021E-08) via HSPs.

2.4 g:Profiler

g:Profiler [1, 2] is a functional annotation tool and associates the combination initiated response to unfolded proteins (p=0.001).

2.5 GFP construct for analysis of protein mistolding/unfolding

Bioinformatics analysis of the gene expression change induced by the combination suggests that the combination is proteotoxic making protein mis/unfolded that subsequently cause initiation of cellular responses to unfolded proteins. To test this effect an engineered melanoma cell line MelJuso^{YFP} was treated with the combination. This cell line becomes fluorescent [3] if unfolded proteins are accumulated. MelJuso^{YFP} cells exposed to the combination were imaged using time-lapse microscopy (IncuCyte FLR) every 2 hour (see Supplementary Movie S1). There was no or very little fluorescent coming out from MelJuso^{YFP}. Also, the known HSP90 inhibitor 17AAG did not show any response (Supplementary Movie S2). However, the positive control Bortezomib, a proteasome inhibitor, caused the cells to become green (Supplementary Movie S3). In conclusion, further investigations (perhaps with more sensitive models) are needed to fully understand the mechanism of action of the combination.

3 Calculations of statistical variability

3.1 Calculation of variability for the first experiment using MACS

During this experiment,13 clinically relevant cytotoxic drugs were used to find out the combination that provide the maximum therapeutic index when treated against model normal DLD-1KRAS/- and cancer cells DLD-1. For this purpose, the fitness value/therapeutic window F studied was defined as difference between survival indices S_n and S_c of normal and cancer cells respectively. Therefore the (population) variance σ_F^2 associated with F is the sum of variances of normal and cancer cells denoted as σ_n^2 and σ_c^2 respectively. More formally:

Case-I:

$$\begin{cases} F = S_n - S_c \\ \sigma_F^2 = \sigma_n^2 + \sigma_c^2 \end{cases}$$

Often each experiment is performed N_r times in same batch. Let $\bar{F}$ denote the mean of fitness values $F(i)$ resulting from N_r repeated experiments for each combination. Similarly, let $\sigma_{\bar{F}}^2$ denote the (population) variance of $\bar{F}$. Then we can write

$$\begin{cases} \bar{F} = \frac{1}{N_r}\sum_{i=1}^{N_r} F(i) = \frac{1}{N_r}\sum_{i=1}^{N_r} S_n(i) - S_c(i) \\ \sigma_{\bar{F}}^2 = \frac{1}{N_r}(\sigma_n^2 + \sigma_c^2) \end{cases}$$

Estimates based on samples available: In practice we need to estimate population variance from available samples. Let $\hat{\sigma}_n^2$ denote the sample variance for normal cell lines and let $\bar{S}_n$ denote the mean survival index for normal cell line. Finally let $S_n(i)$ denote the $i:th$ replicate's survival index for the normal cell line. These statistics are determined using the following standard expressions:

$$\begin{cases} \bar{S}_n = \frac{1}{N_r}\sum_{i=1}^{N_r} S_n(i) \\ \hat{\sigma}_n^2 = \frac{1}{N_r-1}\sum_{i=1}^{N_r}(S_n(i) - \bar{S}_n)^2 \end{cases}$$

Similarly, the corresponding variance estimate $\hat{\sigma}_c^2$ for the cancer cell lines may be calculated as $\hat{\sigma}_c^2 = \frac{1}{N_r-1}\sum_{i=1}^{N_r}(S_c(i) - \bar{S}_c)^2$ where $\bar{S}_c = \frac{1}{N_r}\sum_{i=1}^{N_r} S_c(i)$ Once the estimated $\hat{\sigma}_n^2$ and $\hat{\sigma}_c^2$ have been calculated it is straightforward to calculate sample variance $\hat{\sigma}_{\bar{F}}^2$ as $\hat{\sigma}_{\bar{F}}^2 = \frac{1}{N_r}(\hat{\sigma}_n^2 + \hat{\sigma}_c^2)$

3.2 Calculation of variability for the main experiment (TACS)

In this experiment 6 drugs were used and the pair of cell lines HCT116 and HT29 was regarded as cancer model while another cell line CCRF-CEM was used as normal/reference/toxicity model. Here the fitness F and the associated variance σ_F^2 can be expressed as

Case-II

$$\begin{cases} F = S_n - \frac{S_{c1}+S_{c2}}{2} \\ \sigma_F^2 = \sigma_n^2 + \frac{\sigma_{c1}^2+\sigma_{c2}^2}{4} \end{cases}$$

where σ_{ci}^2 denotes the variance of the survival of cancer cell line i. Now let N_r denote the number of times each experiment is performed in each batch and let N_B denote the number of batches. Therefore mean fitness $\bar{F}$ can be calculated by averaging the individual fitness values F_{ib} as:

$$\bar{F} = \frac{1}{N_B}\sum_{b=1}^{N_B}(\frac{1}{N_r}\sum_{i=1}^{N_r} F_{ib}) = \frac{1}{N_B N_r}\sum_{b=1}^{N_B}\sum_{i=1}^{N_r} F_{ib}.$$

Thus the variance $\sigma_{\bar{F}}^2$ for $\bar{F}$ can be calculated as:

$$\sigma_{\bar{F}}^2 = \frac{1}{N_B N_r}\sigma_F^2 = \frac{1}{N_B N_r}(\sigma_n^2 + \frac{\sigma_{c1}^2 + \sigma_{c2}^2}{4}).$$

Estimates based on samples available: In practice we need to estimate population variance from available samples. This is achieved by the following standard expressions for the normal cells:

$$\begin{cases} \bar{S}_{nb} = \frac{1}{N_r}\sum_{i=1}^{N_r} S_n(i,b) \\ \hat{\sigma}_b^2 = \frac{1}{N_r-1}\sum_{i=1}^{N_r}(S_n(i,b) - \bar{S}_{nb})^2 \\ \hat{\sigma}_n^2 = \frac{1}{N_B}\sum_{b=1}^{B}\hat{\sigma}_b^2 \end{cases}$$

For the cancer cell lines (j=1,2) one has:

$$\begin{cases} \bar{S}_{cj,b} = \frac{1}{N_r}\sum_{i=1}^{N_r} S_{cj}(i,b) \\ \hat{\sigma}_{cj,b}^2 = \frac{1}{N_r-1}\sum_{i=1}^{N_r}(S_{cj}(i,b) - \bar{S}_{cj,b})^2 \\ \hat{\sigma}_{cj}^2 = \frac{1}{N_B}\sum_{b=1}^{B}\hat{\sigma}_{cj,b}^2 \end{cases}$$

Here $\hat{\sigma}_{nb}^2$ denotes the sample variance for normal cell lines, $\bar{S}_{nb}$ denotes the mean survival index in batch b for the normal/reference/toxicity cell line and $S_n(i,b)$ is the corresponding survival index of experiment i in batch b. Finally the the variance estimate $\hat{\sigma}_F^2$ is determined as

$$\hat{\sigma}_F^2 = \frac{1}{N_B N_r}(\hat{\sigma}_n^2 + \frac{\hat{\sigma}_{c1}^2 + \hat{\sigma}_{c2}^2}{4})$$

where $\hat{\sigma}_{cj}^2 = \frac{1}{N_r-1}\sum_{i=1}^{N_r}(S_{cj}(i,b) - \bar{S}_{cj,b})^2$, $S_{cj}(i,b)$ denotes the survival index of cell line j when in batch b, and $\bar{S}_{cj,b}$ denotes the average survival index of cell line j in batch b.

3.3 Calculation of variance for several cancer cell lines

When comparing the mean survival index of 5 CRC cell lines and a normal model, the population variance of this mean survival index is calculated as follows. Let $S_{i,j}$ denote the estimated survival index of CRC cell line i in the jth experiment ($i = 1, 2, ..., N$ and $j = 1, 2, ..., M$). In our case $N = 5$ and $M = 3$. Then the sample mean $\hat{\mu}_S$ of the survival index and its population variance $\sigma_{\hat{\mu}_S}^2$ can be written

$$\begin{cases} \hat{\mu}_S = \frac{1}{N}\sum_{n=1}^{N}(\frac{1}{M}\sum_{j=1}^{M} S_{ij}) = \frac{1}{NM}\sum_{n=1}^{N}\sum_{j=1}^{M} S_{ij} \\ \sigma_{\hat{\mu}_S}^2 = \frac{1}{NM}\sigma_S^2 \end{cases}$$

where σ_S^2 denotes the (population) variance of one single SI measurement.

Estimates based on samples available: In practice we need to estimate the population variance $\sigma^2_{\hat{\mu}_S}$ from available samples. This is achieved by the following standard expressions where $\hat{\mu}_S(i)$ denotes the sample mean based only on the M experimental SI values from the ith cell line:

$$\begin{cases} \hat{\mu}_S(i) = \frac{1}{M}\sum_{j=1}^{M} S_{i,j} \\ \hat{\sigma}^2_{S,i} = \frac{1}{M-1}\sum_{i=1}^{M}(S_{i,j} - \hat{\mu}_S(i))^2 \\ \hat{\sigma}^2_S = \frac{1}{N}\sum_{i=1}^{N}\hat{\sigma}^2_{S,i} \end{cases}$$

Finally the the variance estimate $\hat{\sigma}^2_S$ is determined as

$$\hat{\sigma}^2_S = \frac{1}{NM}\hat{\sigma}^2_S = \frac{1}{N^2M}\sum_{i=1}^{N}\hat{\sigma}^2_{S,i}.$$

4 Performance monitoring of experimental/computational pipeline

Performance of the pipeline was constantly monitored by comparison of survival indices produced with treatment of a combination when replicated within and multiple independent experiments. In the supplementary Figure S11, results are presented for those combinations that were replicated in multiple independent experiments, represented by Gen 0, Gen 1 and so on, and repeated 3 times in an experiment. In the first experiment using MACS (Panel A & B), intra experimental variability resulted after treatment of DLD-1 and DLD-1KRAS/- with five and four drug combinations is represented by standard error of mean (SEM) and gray scale bars represent mean survival in different independent experiments. Apparently no indication of large variability was found (In Panel A the standard deviation (SD) is only 1.1 SI units). For the second (main) experiment (Panel C-D), with already mentioned settings, the maximum SD was 5.25 SI units was calculated when cells lines HCT116, HT29 and CCRF-CEM were treated with combination *Rapamycin,17-AAG, Trichostatin A*. For the combination *Sunitinib, 17-AAG, Afungin, Trichostatin A* the maximum SD was 3.5 SI units. The average SD across all combinations in second (main) experiment is 4.4 SI units suggesting that there is no strong variability. Additionally, the variability observed here is comparable to the variability reported by others in already published work where the end point analysis was also performed with FMCA [4]. The experimental variability observed in our pipeline was also comparable to those published by others when iterative search towards optimal drug combinations was performed, please refer to Figure 3 and supplementary material in [5].

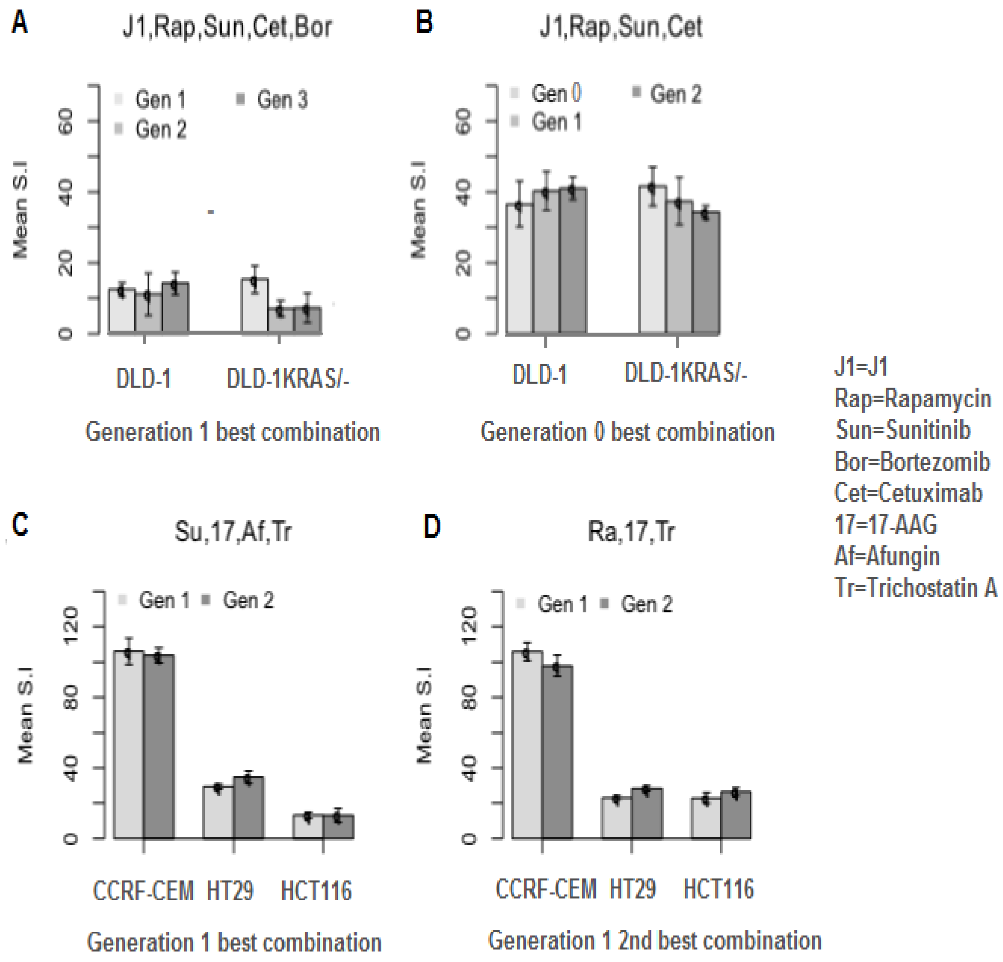

Figure S11: **Performance monitoring of experimental/computational pipeline. Panel A & B**: Survival of DLD-1 and DLD-1KRAS cell lines in multiple experiments when treated with five and four drug combinations replicated three times in an experiment. Intra experimental variability is represented by SEM and gray scale bars represent mean survival of same combination when repeated in multiple experiments. Apparently no indication of large variability is found. **Panel C & D**: Survival of the three cell lines CCRF-CEM, HCT116 and HT29 when treated with different length combinations in multiple independent experiments, only small variability can be found.

References

[1] Reimand J, Kull M, Peterson H, Hansen J, Vilo J. g:Profiler – a web-based toolset for functional profiling of gene lists from large-scale experiments. NAR. 2007;35:193–200.

[2] Reimand J, Arak T, Vilo J. g:Profiler – a web server for functional interpretation of gene lists(2011 update). Nucl Acids Res. 2011;39(Suppl 2):W307–W315.

[3] Benito-Menendez V, Verhoef LGGC, Masucci MG, Dantuma NP. Endoplasmic reticulum stress compromises the ubiquitin-proteasome system. Hum Mol Genet. 2005;14:2787–2799.

[4] Lindhagen E, Nygren P, Larsson R. The Fluorometric microculture cytotoxicity assay. Nat Protoc. 2008;3:1364–1369.

[5] Zinner RG, Barrett BL, Popova E, Damien P, Volgin AY, Gelovani JG, et al. Algorithmic guided screening of drug combinations of arbitrary size for activity againt cancer cells. Mol Cancer Ther. 2009;8(3):521–532.

Supplementary Methods II:
In vitro discovery of promising anti-cancer drug combinations using maximization of a therapeutic index

M. Kashif[1], C. Andersson[1], S. Hassan[1], H. Karlsson[1], W. Senkowski[1], M. Fryknäs[1], P. Nygren[2], R. Larsson[1], M.G. Gustafsson[1,*]

[1]Uppsala University, Dept. Medical Sciences Cancer Pharmacology and Computational Medicine, Academic Hospital, 751 85 Uppsala, Sweden

[2]Uppsala University, Dept of Radiology, Oncology and Radiation Science, Academic Hospital, SE-751 85 Uppsala, Sweden.

*Corresponding author: Mats.Gustafsson@medsci.uu.se

Brief Description:

This supplement includes Beckman Coulter® Biomek 2000 cherry picking programme and Matlab® code for prediction of IC10 and IC20 values from concentration response data.
Supplementary Figure S12: Cell plate layout.

1-Beckman Coulter® Biomek 2000 cherry picking programme

Plate layout

Cell Plate Layout

	1	2	3	4	5	6	7	8	9	10	11	12
A		M	E	D	I	U	M		O	N	L	Y
B	M	C4	C8	C10	C13	C	C13	C2	C6	D1.X1	M	M
C	E	C5	C13	C11	C7	O	C9	C2	C7	D2.X1	E	E
D	D	C3	C8	C11	C3	N	C11	C8	C1	D3 X1	D	D
E	I	C6	C6	C10	C1	T	C3	C4	C4	D4 X1	I	I
F	U	C5	C10	C14	C12	R	C12	C7	C2	D5 X1	U	U
G	M	C12	C5	C1	C14	OL	C14	C9	C9	D6 X1	M	M
H		M	E	D	I	U	M		O	N	L	Y

C=Combination,D=Drug, X=Dose
B6 to G6=Cells with medium only, used as control
B11 to G11= Wells with medium only, used as blank
B10 to G10= Wells with all drugs with single dose
B11 to G11= Wells with all drugs with six time dose

Figure S12. **Cell plate layout:** Total of 14 combinations was fitted on this plate with triplicates at random places. C stands for combination proceeded by combination number. Controls without any treatment are at column 6 and colored green. Empty control with medium only is column 1 and 11. Outer rows and columns were excluded from experiments to avoid (false) effects that could be produced in these wells by hydration.

#This script is written for Beckman Coulter® Biomek 2000 robotic platform (Biomek 2000) by #using BioScript Pro.

Terms used

#1- Biomek 2000 robotic arm= Robotic arm of Biomek 2000 where tips attach to, and that arm moves #three dimensionally from source to destination.

#2- Biomek 2000 work surface= The space of Biomek 2000 where different labwares are placed.

#3-Labwares= Various wet-lab equipment like plates, racks, tubes etc. that can be used with Biomek #2000.

#X, Y and Z coordinates= Viewing Biomek 2000 from front; X is the left-to-right axis across the #work surface, Y is the front-to-back axis and Z is up-to-down axis

#**Description of Programme**

#This program transfers desired volumes (>2µl <100 µl) of drugs from source well to destination well. #Biomek 2000 work surface is divided in to two rows that are further subdivided in to 6 locations each. #Labwares are placed on these locations.

#Each drug transfer starts with attachment of new tip on robotic arm then aspiration of desired volume #from source labware and finally dispensing in to destination well. Appendorf tubes were used as source #labware and destination labwares were Nunc® 96 well plates with flat or v-shaped bottom.

```
Flag avoid 1

# Initialize labwares and their location on work surface
  set source "Microfuge 24"       # Name of source labware to hold appendorf tubes
  set sourcedrug  B3              # Position of first "Microfuge 24" on work surface
                                  #used for drugs
  set sourcemedium B1             # Position of second "Microfuge 24" on work surface
                                    #used for PBS
  set dest "96-well flat"         # Name of destination labware
  set destination B2              # Position of "96-well flat" on work surface

# Specify limits how deep tip can go in labwares
  set srcheight 0.05;   # Aspirate drug from 5% above bottom of source labware
  set dstheight 0.6 ;   # Dispense 60% above bottom of destination labware

# Aspiration delay. It is time that after aspiration of a drug; a tip will remain inside (liquid) drug well to
#make aspiration accurate.
  set Adelay 0.5
```

```
# Amount of blow out air after dispensing drug. This air will help to drop any liquid attached to tip after
#dispension.
 set blowoutair 15

# Extract coordinates of labwares and then calculates how deep tip can move in them.
# Extract coordinates of labwares
  set src(SrcHt) [GetVal labware $source c_top]        #Height of source  labware
  set src(SrcDp) [GetVal labware $source c_depth]   #Depth  of source  labware

  set dst(DstHt) [GetVal labware $dest c_top]           #Height of destination  labware
  set dst(DstDp) [GetVal labware $dest c_depth]      #Depth of destination labware
# Calculation of Z-axis values (How deep tips will go in the wells)
  set srcZ [expr $src(SrcHt) - $src(SrcDp) + ( $srcheight * $src(SrcDp)) ]
  set dstZ [expr $dst(DstHt) - $dst(DstDp) + ( $dstheight * $dst(DstDp)) ]

# Get the current tool attached to robotic arm and steps per microliter volume
 set tool [GetVal system curtool pTool]                        # current tool attached to robotic arm
 set step [GetVal tools $tool steps_per_microliter]          # How many steps transfer motor will make per
                                                                         # microliter

# Prepare transfer motor for aspiration
 set move [expr $step * $tvolume]                    #Move transfer motor

# Specify destination plate layouts, see Figure S15 for details.
# Each combination is replicated on destination plate in wells those were selected (once) randomly at the
#beginning of whole set of experiments. Their positions are defined below in the array randomwells.

#Note about array definition:
# Array in BioScript Pro is defined by as
# set arrayname(index) value.
#or
# set arrayname  list, Number of elements in the list should be even and every odd element is the index
# while even is  value of that index

# Specify positions of replicate wells on destination plate.
 set randomwells(1) 38; set randomwells(2) 21; set randomwells(3) 25; set randomwells(4) 6
 set randomwells(5) 35; set randomwells(6) 13; set randomwells(7) 32; set randomwells(8) 26
 set randomwells(9) 18; set randomwells(10) 15; set randomwells(11) 28; set randomwells(12) 27
 set randomwells(13) 1; set randomwells(14) 37; set randomwells(15) 40; set randomwells(16) 46
 set randomwells(17) 29; set randomwells(18) 23; set randomwells(19) 48; set randomwells(20) 7
 set randomwells(21) 22; set randomwells(22) 34; set randomwells(23) 14; set randomwells(24) 11
 set randomwells(25) 47; set randomwells(26) 8; set randomwells(27) 2; set randomwells(28) 20
 set randomwells(29) 12; set randomwells(30) 42; set randomwells(31) 24; set randomwells(32)41
 set randomwells(33) 45; set randomwells(34) 30; set randomwells(35) 3; set randomwells(36) 19
```

```
 set randomwells(37) 10; set randomwells(38) 17; set randomwells(39) 33; set randomwells(40) 43
 set randomwells(41) 36; set randomwells(42) 9; set randomwells(43) 16; set randomwells(44) 5
 set randomwells(45) 4; set randomwells(46) 39; set randomwells(47) 31; set randomwells(48) 44

# Destination plate layout
 set destinationwells(1) B2; set destinationwells(2) B3; set destinationwells(3) B4; set destinationwells(4)
B5
 set destinationwells(5) B7; set destinationwells(6) B8; set destinationwells(7) B9; set destinationwells(8)
B10
 set destinationwells(9) C2; set destinationwells(10) C3; set destinationwells(11) C4; set
destinationwells(12) C5
 set destinationwells(13) C7; set destinationwells(14) C8; set destinationwells(15) C9; set
destinationwells(16) C10
 set destinationwells(17) D2; set destinationwells(18) D3; set destinationwells(19) D4; set
destinationwells(20) D5
 set destinationwells(21) D7; set destinationwells(22) D8; set destinationwells(23) D9; set
destinationwells(24) D10
 set destinationwells(25) E2; set destinationwells(26) E3; set destinationwells(27) E4; set
destinationwells(28) E5
 set destinationwells(29) E7; set destinationwells(30) E8; set destinationwells(31) E9; set
destinationwells(32) E10
 set destinationwells(33) F2; set destinationwells(34) F3; set destinationwells(35) F4; set
destinationwells(36) F5
 set destinationwells(37) F7; set destinationwells(38) F8; set destinationwells(39) F9; set
destinationwells(40) F10
 set destinationwells(41) G2; set destinationwells(42) G3; set destinationwells(43) G4; set
destinationwells(44) G5
 set destinationwells(45) G7; set destinationwells(46) G8; set destinationwells(47) G9; set
destinationwells(48) G10

# Set source plate format (Each well containing different drugs)
 set sourcewells(1) A1; set sourcewells(2) A2; set sourcewells(3) A3; set sourcewells(4) A4; set
sourcewells(5) A5; set sourcewells(6) A6
 set sourcewells(7) B1; set sourcewells(8) B2; set sourcewells(9) B3; set sourcewells(10) B4 ; set
sourcewells(11) B5; set sourcewells(12) B6
 set sourcewells(13) C1; set sourcewells(14) C2; set sourcewells(15) C3; set sourcewells(16) C4 ; set
sourcewells(17) C5; set sourcewells(18) C6
 set sourcewells(19) D1; set sourcewells(20) D2

#Specify source wells containing PBS.
# It is required to reach same final volume in destination wells.  Every destination well has 170microLiter
#of cell suspension before drug transfer. In case of two drug combination 10µl of drugs will be added in
#destination well to reach final volume of 200µl, it is required to add 20µl of PBS in it.
 set pbswells(1) A6; set pbswells(2) B6; set pbswells(3) C6
```

```
 set pbswells(4) D6; set pbswells(5) E6; set pbswells(6) F6
 set pbswells(7) A5
vdi_create vdi1 "vdi_value"

# Specify excel sheet name
extref_init "EXCEL|Sheet1"

# No of wells in an experiments
  set noofwells [extref_get "EXCEL|Sheet1|R1C20" ]

# Transfer volume in microLiters
  set tvolume [extref_get "EXCEL|Sheet1|R1C21" ]

# Maximum number of drugs in a combination
  set maxdrugs [extref_get "EXCEL|Sheet1|R1C22" ]


##### Code to extract source and destination location from spread sheet and perform desired transfers

# Loop to read the drug numbers from the excel file and then move to transfer the drugs
for {set wells 0} {($wells < $noofwells)} {incr wells} {
    set colno 1
    set drugnumber [extref_get "EXCEL|Sheet1|R[expr $wells + 1]C$colno" ]
        while { ( [string index $drugnumber 0] != "M") &&  ( $colno < maxdrugs )}  {

          # Instructions for Aspiration

        # Move robot arm to source location
           Move Abs [Coord $sourcedrug $sourcewells([string trim $drugnumber])] 55
        #Aspirate blow out air
          Move Rel T $blowoutair
        # Move the tip down in source well
          Move Abs Z $srcZ
        # Aspirate
         Move Rel T $move
       # Keep tip in the source well for some time
        Delay $Adelay
      # Move robot arm and tip out of source well
       Move Abs Z $src(SrcHt)

        # Dispense instructions

       #Move to destination location
          Move Abs [Coord $destination $destinationwells($randomwells([expr $wells + 1]))] 50
```

```
    #Move tip in the destination well
        Move Abs Z $dstZ
    #Dispense
        Move Rel T [expr $move * -1]
    #Blow out extra air
        Move Rel T [expr $blowoutair * -1]
   # Tip touch
       Move Rel X 3
       Move Rel X -3
  # Move out of destination well
      Move Abs Z $dst(DstHt)

   # Replace the Tip
      Tip attach save

 # Increment and read next drug from next cell of spread sheet
   incr colno

   set drugnumber [extref_get "EXCEL|Sheet1|R[expr $wells + 1]C$colno" ]

}

if { $colno < maxdrugs } { #If PBS need to be added in the well

#Volume of PBS that will be added in the well
 set pbsvolume [string index $drugnumber 1][string index $drugnumber 2]
 set pbsvolume [expr int($pbsvolume)]
 set totalpbs [extref_get "EXCEL|Sheet1|R[expr $wells+1]C[expr $colno+1 ]" ]

#Aspiration preparation
set pbsMove  [expr $step * $pbsvolume]


   # Aspirate
     Move Abs [Coord $sourcemedium $pbswells([expr ($totalpbs/625) + 1])] 55
     Move Rel T $blowoutair
     Move Abs Z $srcZ
     Move Rel T  $pbsMove
     Delay $Adelay
     Move Abs Z $src(SrcHt)

   # Dispense
     Move Abs [Coord $destination $destinationwells( $randomwells([expr $wells + 1]))] 50
     Move Abs Z $dstZ
```

```
        Move Rel T [expr $pbsMove * -1]
        Move Rel T [expr $blowoutair * -1]
        Move Rel X 3
        Move Rel X -3
        Move Abs Z $dst(DstHt)

      # Replace the Tip
        Tip attach save
    }
}
extref_end_all
vdi_destroy vdi1
```

2- Matlab® Code for prediction of IC10, IC20 values from concentration response data.

Description:

1 % This code predicts the IC20 and IC10 values from concentration response data by fitting hill regression
2 %model. Graphs of fitted curve and IC10 and IC20 values are saved/written to a file (argument of the
3 %analyze function). The input of this program dose response data in the form of text file exported from
4 %graphpad prism®, present in the same directory that contain Matlab® program.
5 %More detailed description of how calculations are made is below:
6 %In order to estimate the IC20 values from dose response curve of selected drugs with cancer
7 %cell lines (DLD-1 for pilot experiment and HCT116 for main experiment) the following
8 %analysis was performed using
9 %the classical Hill model, here described as
10

$$\%E = \frac{c^h}{c^h + (c_{50})^h}$$

11 %where E is the predicted drug effect, c is the drug concentration and c_{50} is the IC50 value, and
12 %the Hill coefficient h determines the slope of dose response. The two parameters c_{50} and h
13 %were fitted by using a nonlinear least-squares-fitting by means of the built-in Matlab®
14 %function fit using default settings (which means employment of a built-in Trust-Region
15 %method) together with the constraints that c_{50} and h are non-negative.
16 %After estimating c_{50} and h using dose-response data, the IC20 value was determined from the
17 % inverse Hill equation as (please see line 142 of code)
18
19 $\%c = c_{50}(\frac{1-E}{E})^{(\frac{1}{h})}$
20
21 %To determine the IC20 value we used E=0.8.

```
analyze(“results.csv”)

function analyze(varargin)
% analyze
% Computes concentrations of SI% effect 10 and 20 from txt-files
% exported from Graphprad Prism. Result is stored in "results.csv".
%
% analyze(filename)
% Stores result in "filename" in the current. NB: Avoid .txt ending.

if nargin == 0
   output = 'results.csv';
else
   output = varargin{1};
end

files = dir('*.txt');
nFile = length(files);

% fid of file used to store result
fid = fopen(output, 'w');

for iFile = 1:nFile
   process(files(iFile).name, fid);
end

fclose(fid);
end

function process(file, fid)
   % importfile creates variables "data" containing numeric
   % data and "textdata" containing the headers, i.e. cell line
   importfile(file);

   % Pad data to original number of columns (import ignores trailing
   % empty columns)

   nPadColumn = length(textdata) - size(data,2);
   nRow = size(data,1);
   data = [data, NaN(nRow, nPadColumn)];

   % Fit curves
   cellLines = setdiff(unique(textdata), textdata{1});
   nCell = length(cellLines);
   conc = data(:,1);
   for iCell = 1:nCell

      si = data(:,strcmp(cellLines{iCell}, textdata));
      [h, ic50] = hillfit(conc, si);


       % The files are given a meaningful name(drugname + cell line) and
       % saved in tiff format.
```

```
        title(['filename =' file , '   Cell line=', cellLines{iCell} ])
        restChars = {'\','/'};
        saveas(gcf, regexprep(['filename =' file , 'Cell line=', cellLines{iCell}], restChars,'-'),'tiff'   )

        ic10 = invhill(h, ic50, .9);
        ic20 = invhill(h, ic50, .8);
        res(1,iCell) = ic10;
        res(2,iCell) = ic20;
    end

    % Print out result
    fprintf(fid, file);
    for iCell = 1:nCell
        fprintf(fid, ',%s', cellLines{iCell});
    end
    fprintf(fid, '\n');

    fprintf(fid, 'IC10');
    for iCell = 1:nCell
        fprintf(fid, ',%d', res(1, iCell));
    end
    fprintf(fid, '\n');

    fprintf(fid, 'IC20')
    for iCell = 1:nCell
        fprintf(fid, ',%d', res(2, iCell));
    end
    fprintf(fid, '\n');
end

function [h, ic50] = hillfit(conc, si)

    % Reformat data for curvefit
    nColumn = size(si, 2);
    x = repmat(conc, nColumn,1);
    y = si(:);
    filter = not(isnan(y));
    x = x(filter);
    % Rescale SI% to SI
    y = y(filter)/100;

    f = fittype('c^h/(c^h + x^h)');
    s = fitoptions('Method','NonlinearLeastSquares',...
              'Lower', [0, 0],...
              'Startpoint',[mean(conc) 1]);

    regression = fit(x, y, f, s);

    % The semilog graphs are produced and then curve is fitted.
```

```
    %figure; Uncomment to enable popup of the figures
     semilogx(x,y);
       hold on
        plot(regression,x,y)
       hold off

    coeffs = coeffvalues(regression);
    ic50 = coeffs(1);
    h = coeffs(2);

end

function ic = invhill(h, ic50, f)
    ic = ic50*((1-f)/f)^(1/h);
end

function importfile(fileToRead1)
%IMPORTFILE(FILETOREAD1)
%  Imports data from the specified file
%  FILETOREAD1:  file to read

% Import the file
newData1 = importdata(fileToRead1);

% Create new variables in the base workspace from those fields.
vars = fieldnames(newData1);
for i = 1:length(vars)
    assignin('caller', vars{i}, newData1.(vars{i}));
end
```

Supplementary Methods Part III: In vitro discovery of promising anti-cancer drug combinations using maximization of a therapeutic index

M. Kashif[1], C. Andersson[1], S. Hassan[1], H. Karlsson[1], W. Senkowski[1], M. Fryknäs[1], P. Nygren[2], R. Larsson[1], M.G. Gustafsson[1,*]

[1]Uppsala University, Dept. Medical Sciences Cancer Pharmacology and Computational Medicine, Academic Hospital, 751 85 Uppsala, Sweden

[2]Uppsala University, Dept of Radiology, Oncology and Radiation Science, Academic Hospital, SE-751 85 Uppsala, Sweden.

*Corresponding author: Mats.Gustafsson@medsci.uu.se

Brief Description:

This supplement describes t-test for therapeutic synergy.

When testing for therapeutic synergy we are whether the difference between two differences are equal or not (i.e. if the therapeutic index for the combination is equal to that of the best single drug). Assuming all measurements are distributed Normal with equal variance σ^2 we can describe the hypothesis test as testing the null hypothesis $H_0\colon \mu_1 - \mu_2 = 0$ vs the alternative $H_1\colon \mu_1 - \mu_2 > 0$ where μ_1, μ_2 are the true TI values of combination and best single respectively. The TI comparison thus corresponds to $Y_1 - Y_2$ where

$$Y_1 = \bar{X}_1 - \bar{X}_2$$
$$Y_2 = \bar{X}_3 - \bar{X}_4$$

and $\bar{X}_i$ is the mean over a set of replicates for the different cell lines and combo and single respectively and $\mu_i = E[Y_i]$.

Test

Under the assumption of equal variance of the measurements we find that $Y_1 - Y_2 \sim Normal\left(\mu_1 - \mu_2, \sigma\sqrt{\frac{1}{n_1} + \frac{1}{n_2} + \frac{1}{n_3} + \frac{1}{n_4}}\right)$, so

$$\frac{Y_1 - Y_2}{\sqrt{\sigma^2\left(\frac{1}{n_1} + \frac{1}{n_2} + \frac{1}{n_3} + \frac{1}{n_4}\right)}} \sim Normal(0,1) \quad (*)$$

when H_0 is true. However, σ^2 is unknown so it cannot be used as a test statistic, and we will need to use some t-statistic.

Now, a variable T is t-distributed with v degrees of freedom if $T = \frac{Z}{\sqrt{V/v}}$ where Z is Normal(0,1), and V is χ^2_v. Given that

$$\frac{\sum_i (X_i - \bar{X})^2}{\sigma^2} \sim \chi^2_{n-1}$$

when n is the number of observations X_i all of which are iid Normal(μ, σ) note that the nominator is simply a constant times the sample variance, i.e. $(n-1)S^2$. Now consider a pooled sample variance obtained from our four sets of measurements above. Each set of replicates will have sample variance S_i^2, based on n_i replicates and we can compute a pooled estimate as

$$S_p^2 = \frac{\sum_i (n_i - 1) S_i^2}{\sum_i (n_i - 1)}$$

From the above and that a sum of independent chi-2 distributed stochastic variables is also chi2-distributed one has in our case that a sum of independent chi-2 distributed stochastic variables is also chi2-distributed one has in our case $\frac{\sum_i (n_i - 1)}{\sigma^2} S_p^2 \sim \chi^2_{n_1+n_2+n_3+n_4-4}$. Dividing (*) with the left hand side shows that

$$t = \frac{Y_1 - Y_2}{\sqrt{\frac{\sum_{j=1}^4 (n_j - 1) S_j^2}{\sum_{j=1}^4 (n_j - 1)}} \sqrt{\left(\frac{1}{n_1} + \frac{1}{n_2} + \frac{1}{n_3} + \frac{1}{n_4}\right)}} = \frac{Y_1 - Y_2}{\sqrt{S_p^2 \left(\frac{1}{n_1} + \frac{1}{n_2} + \frac{1}{n_3} + \frac{1}{n_4}\right)}}$$

is t-distributed with $n_1 + n_2 + n_3 + n_4 - 4$ degrees of freedom. Thus the pooled variance S_p^2 can be used together with the difference $Y_1 - Y_2$ to determine a test statistic which is t-distributed with $n_1 + n_2 + n_3 + n_4 - 4$ degrees of freedom from which a p-value for the stated H_0 above can be computed.
