# Supplementary material for: In vitro discovery of promising anti-cancer drug combinations using iterative maximisation of a therapeutic index
Source: Sci Rep. 2015 Sep 22;5:14118. doi: 10.1038/srep14118 (PMC4585751; doi:10.1038/srep14118)
Supplement: Supplementary Data [file srep14118-s3.pdf]

Supplementary Information:
In vitro discovery of promising anti-cancer drug combinations using maximization of a therapeutic index

M. Kashif[1], C. Andersson[1], S. Hassan[1], H. Karlsson[1], W. Senkowski[1], M. Fryknäs[1] P. Nygren[2], R. Larsson[1], M.G. Gustafsson[1,*]
[1]Uppsala University, Dept of Medical Sciences, Cancer Pharmacology and Computational Medicine, Academic Hospital, SE-751 85 Uppsala, Sweden. [2]Uppsala University, Dept of Radiology, Oncology and Radiation Science, Academic Hospital, SE-751 85 Uppsala, Sweden.

1 Brief Description

Supplementary Tables S1-S8. Table S1: A set of 13 clinical/experimental drugs used in the iterative search using the MACS algorithm (pilot experiment). Table S2: Results of generation 0 of pilot experiment. Table S3: Results of generation 1 of pilot experiment. Table S4: Results of generation 2 of pilot experiment. Table S5: Results of generation 3 of pilot experiment.
Table S6: Results of generation 0 of main experiment. Table S7: Results of generation 1 of main experiment. Table S8: Results of generation 2 of main experiment.
Supplementary Figures S1-S10. Figure S1: Optimization overview of all iterations of pilot experiment. Figure S2: Factorial concentration-response and TS (therapeutic synergy) study of combination (Sunitinib, 17-AAG, Afungin, Trichostatin A). Figure S3: Factorial concentration-response study of combination (Sunitinib, 17-AAG, Afungin, Trichostatin A) in patient cells. Figure S4: Factorial concentration-response and TS study of combination (Rapamycin, 17-AAG, Trichostatin A). Figure S5: Factorial concentration-response study of combination (Rapamycin, 17-AAG, Trichostatin A) in patient cells. Figure S6: Factorial concentration-response and TS study of combination (Rapamycin, 17-AAG). Figure S7: Factorial concentration-response study of combination (Rapamycin, 17-AAG) in patient cells. Figure S8: Factorial concentration-response and TS study of combination (Sunitinib, 17-AAG, Afungin). Figure S9: Factorial concentration-response study of combination (Sunitinib, 17-AAG, Afungin) in patient cells. Figure S10: Concentration-response study of combination (17-AAG, Afungin, Trichostatin A) in patient cells performed at five different concentrations.

2 Pilot study

In a pilot study, we evaluated MACS[1] and evolved towards TACS, using an isogenic pair of human CRC cell lines DLD-1 and DLD-1KRAS/- which are isogenic except that DLD-1 expresses mutated KRAS allele whereas in DLD-1KRAS/- it is knocked out. The fitness criterion used was the survival of therapeutically more challenging DLD-1 relative to the survival of the "repaired" DLD-1KRAS/-. Thus, the optimal combination would have no effect on DLD-1KRAS/- but completely eradicate DLD-1. Drugs used in this experiment are listed in Table S1.

Table S1: A set of 13 clinical/experimental drugs used in the iterative search using the MACS algorithm (pilot experiment).

Sr.	Drug	Suggested Activity	Obtained from	Stock Conc.	Final Conc.
1	5 Fluorouracil	active anti-neoplastic agent[2], for decades main stay for colorectal cancer treatment	Sigma-Aldrich Sweden AB	400µM	10µM
2	Doxorubicin	topoisomerase II poison[3]	Sigma-Aldrich Sweden AB	20µM	0.5µM
3	Cetuximab	inhibitor the EGFR/MAPK pathway[4]	Apotekt	1000µg/mL	25µg/mL
4	Docetaxel	antimicrotubule agent [5]	Sigma-Aldrich Sweden AB	0.4µM	0.01µM
5	J1	Prodrug of melphalan[6]	Oncopeptide AB, Sweden	54.40 µM	1.36µM
6	Sunitinib	tyrosine kinase inhibitor[7]	Lc Laboratories USA	134.8µM	3.37µM
7	Erlotinib	epidermal growth factor receptor (EGFR), tyrosine kinase inhibitor[8]	Lc Laboratories USA	1023.6µM	25.6µM
8	Bortezomib	proteasome inhibitor[9]	Lc Laboratories USA	0.898µM	0.0225µM
9	Rapamycin	blocks the activation mTOR (mammalian target of rapamycin) [10]	Sigma-Aldrich Sweden AB	131.6µM	3.29µM
10	Acriflavinium	inhibits HIF-1 dimerization, tumor growth, and vascularization[11]	Sigma-Aldrich Sweden AB	56µM	1.4µM
11	Trichostatin A	histone deacetylase inhibitor[12]	Sigma-Aldrich Sweden AB	8.4µM	0.21µM
12	Vivolux 60		Experimental drug	37.6µM	0.94µM
13	AAG 17	Heat shock protein(Hsp) inhibitor, causing G1 arrest and decreased DNA synthesis[13]	Lc Laboratories USA	27.2µM	0.68µM

Pilot study was initialized by generation 0 (Table S2) of MACS with 14 randomly selected combinations of arbitrary size. Largest combination consisted of 11 anti-cancer drugs while the smallest combination contained 3 drugs only. A combination *J1, Rapamycin, Sunitinib and Cetuximab* was found top ranked with therapeutic index 5. Generation 1 of pilot study was created as per MACS algorithm and found that top two combinations (fitness ranked 1 and 2 in Table S3) have therapeutic indices very similar 2.9 and 2.8, respectively. As per MACS, combination with highest therapeutic index 2.9 was selected and generation 2 was created. In generation 2 (Table S4) again top ranked combination (therapeutic index 0.9) was found to have 4 other combinations ranked 2-5 with very little difference in their therapeutic indices (1, 1.2, 1.7 and 2 SI units only). These differences were within noise range of top ranked combination therefore these all were probably equal hence should be candidate for parent of new generation. By taking experimental variability into account (a step towards TACS) all these combinations were considered equal and two combinations at rank 3 and 4 with least number of drugs (4) were selected as final candidates of parent of generation 3. Among them, a combination (J1, Rapamycin, Sunitinib and Bortezomib) at rank 3 was selected due to higher rank. In generation 3 (Table S5), search converged to a combination *J1, Sunitinib and Bortezomib* as the most promising. In Figure S1, selected combinations in each generation (0-3) are assigned the different colors blue (gen 0), cyan (gen 1), yellow (gen 2), and green (gen 3), respectively. It can be noted that only one combination for each generation was used as parent of next generation.

Table S2: Results from generation 0 in pilot experiment. The columns "SI(kras) and SI(wt)" show survival indices for treatments of DLD-1KRAS/- and DLD-1 cells, respectively. The treatments used are specified in column labeled "Combinations". Column "Therapeutic Index" contains the difference (SI(kras)- SI(wt)) between two SI values and column named "Fitness rank" specifies the rank of every combination on basis of its therapeutic index. Top ranked combination *J1, Rapamycin, Sunitinib and Cetuximab* was selected as parent of next generation.

Sr. No.	S.I. (kras)	S.I. (wt)	Therapeutic Index	Fit. ranks	No. drugs	Combinations
1	41.6	36.6	5	1	4	J1 - RAPAMYCIN - SUNITINIB - CETUXIMAB
2	5.1	1.4	3.7	2	7	DOXOROBICIN - DOCETAXEL - BORTEZOMIB - ACRIFLAVINIUM - TRICHOSTATIN - VIVOLUX 60 - AAG 17
3	5.7	5.2	0.5	4	7	J1 - 5FU - RAPAMYCIN - SUNITINIB - ERLOTINIB - CETUXIMAB - TRICHOSTATIN
4	0.7	1	-0.3	5	11	5FU - VIVOLUX 60 - ERLOTINIB - CETUXIMAB - J1 - BORTEZOMIB - DOXOROBICIN - AAG 17 - DOCETAXEL - TRICHOSTATIN - ACRIFLAVINIUM
5	7.4	6.2	1.2	3	5	J1 - DOXOROBICIN - TRICHOSTATIN - VIVOLUX 60 - AAG 17
6	4.9	9	-4.1	6	5	J1 - 5FU - BORTEZOMIB - ERLOTINIB - RAPAMYCIN
7	9.6	16.6	-7	8	6	J1 - BORTEZOMIB - 5FU - RAPAMYCIN - DOXOROBICIN - SUNITINIB
8	57.6	62.1	-4.5	7	3	5FU - CETUXIMAB - ERLOTINIB
9	5.7	13.6	-7.9	9	5	J1 - BORTEZOMIB - 5FU - RAPAMYCIN - DOXOROBICIN
10	25.4	43.2	-17.8	11	5	5FU - RAPAMYCIN - SUNITINIB - ERLOTINIB - CETUXIMAB
11	28.9	47.9	-19	13	5	5FU - CETUXIMAB - ERLOTINIB - RAPAMYCIN - SUNITINIB
12	35.7	51.4	-15.7	10	4	J1 - ERLOTINIB - RAPAMYCIN - CETUXIMAB
13	17.3	35.4	-18.1	12	6	J1 - 5FU - CETUXIMAB - ERLOTINIB - RAPAMYCIN - SUNITINIB
14	30	50.9	-20.9	14	4	J1 - 5FU - RAPAMYCIN - ERLOTINIB

Table S3: Results from generation 1 in pilot experiment. Top ranked combination *J1, Rapamycin, Sunitinib, Cetuximab and Bortezomib* was selected as parent of next generation. It can be noted that top two combinations have therapeutic indices very similar, 2.9 and 2.8.

Sr. No.	S.I. (kras)	S.I. (wt)	Therapeutic Index	Fit. ranks	No. drugs	Combinations
1	15.3	12.4	2.9	1	5	J1 - RAPAMYCIN - SUNITINIB - CETUXIMAB - BORTEZOMIB
2	31.9	29.1	2.8	2	5	J1 - RAPAMYCIN - SUNITINIB - CETUXIMAB - ACRIFLAVINIUM
3	8.2	8.7	-0.5	3	5	J1 - RAPAMYCIN - SUNITINIB - CETUXIMAB - TRICHOSTATIN
4	4.9	6	-1.1	4	5	J1 - RAPAMYCIN - SUNITINIB - CETUXIMAB - VIVOLUX 60
5	48.6	52.6	-4	6	3	J1 - RAPAMYCIN - SUNITINIB
6	23.7	28.5	-4.8	7	5	J1 - RAPAMYCIN - SUNITINIB - CETUXIMAB - AAG 17
7	37.4	40.3	-2.9	5	4	J1 - RAPAMYCIN - SUNITINIB - CETUXIMAB
8	64.4	72.7	-8.3	8	3	J1 - RAPAMYCIN - CETUXIMAB
9	31.8	43	-11.2	10	5	J1 - RAPAMYCIN - SUNITINIB - CETUXIMAB - DOXOROBICIN
10	29	40.7	-11.7	11	5	J1 - RAPAMYCIN - SUNITINIB - CETUXIMAB - ERLOTINIB
11	33.4	45.9	-12.5	12	5	J1 - RAPAMYCIN - SUNITINIB - CETUXIMAB - DOCETAXEL
12	47.5	57.1	-9.6	9	3	RAPAMYCIN - SUNITINIB - CETUXIMAB
13	49.7	70.5	-20.8	13	3	J1 - SUNITINIB - CETUXIMAB
14	26.8	54.4	-27.6	14	5	J1 - RAPAMYCIN - SUNITINIB - CETUXIMAB - 5FU

Table S4: Results from generation 2 in pilot experiment. In this generation top ranked combination has therapeutic index 0.9 and it was found that differences of top ranked and other 4 combinations ranked 2-5 are very small (1, 1.2, 1.7 and 2 SI units only). These differences were within noise range of top ranked combination therefore these all combination(1-5) were probably equal hence should be candidate for parent of new generation. Column "Thr.Ind.-Std" contains difference between top ranked therapeutic index and its standard deviation, this information is helpful in determining that how many combinations are within one standard deviation of top ranked combination (therefore considered to perform the same). A column "Next Gen. candidate" indicates which combinations were selected as candidate for parent of next generation. Therefore by taking experimental variability into account (a step towards TACS) all these combinations were considered equal and two combinations at rank 3 and 4 with least number of drugs (4) were selected as final candidates of parent of generation 3. Among them combination *J1, Rapamycin, Sunitinib and Bortezomib* at rank 3 was selected due to higher rank.

Sr. No.	S.I. (kras)	S.I. (wt)	Therapeutic Index	Fit. ranks	Thr.Ind. -Std	Next Gen. candidate	No. drugs	Combinations
1	12.8	13.1	-0.3	3		Yes	4	J1 - RAPAMYCIN - SUNITINIB - BORTEZOMIB
2	18.2	17.3	0.9	1	-1.24	Yes	6	J1 - RAPAMYCIN - SUNITINIB - CETUXIMAB - BORTEZOMIB - ACRIFLAVINIUM
3	19.5	19.6	-0.1	2		Yes	6	J1 - RAPAMYCIN - SUNITINIB - CETUXIMAB - BORTEZOMIB - AAG 17
4	7.8	8.6	-0.8	4		Yes	4	J1 - RAPAMYCIN - CETUXIMAB - BORTEZOMIB
5	8.4	9.5	-1.1	5		Yes	6	J1 - RAPAMYCIN - SUNITINIB - CETUXIMAB - BORTEZOMIB - 5FU
6	8.4	10.4	-2	8		No	6	J1 - RAPAMYCIN - SUNITINIB - CETUXIMAB - BORTEZOMIB - DOXOROBICIN
7	7.6	9.4	-1.8	7		No	6	J1 - RAPAMYCIN - SUNITINIB - CETUXIMAB - BORTEZOMIB - DOCETAXEL
8	1.7	3.3	-1.6	6		No	6	J1 - RAPAMYCIN - SUNITINIB - CETUXIMAB - BORTEZOMIB - TRICHOSTATIN
9	10.2	14.3	-4.1	11		No	4	J1 - SUNITINIB - CETUXIMAB - BORTEZOMIB
10	7.1	11.1	-4	10		No	5	J1 - RAPAMYCIN - SUNITINIB - CETUXIMAB - BORTEZOMIB
11	0.8	3.5	-2.7	9		No	6	J1 - RAPAMYCIN - SUNITINIB - CETUXIMAB - BORTEZOMIB - VIVOLUX 60
12	18.4	25.0	-6.6	13		No	4	RAPAMYCIN - SUNITINIB - CETUXIMAB - BORTEZOMIB
13	34.2	41.0	-6.8	14		No	4	J1 - RAPAMYCIN - SUNITINIB - CETUXIMAB
14	11.2	17.2	-6	12		No	6	J1 - RAPAMYCIN - SUNITINIB - CETUXIMAB - BORTEZOMIB - ERLOTINIB

Table S5: Results from generation 3 in pilot experiment. In Generation 3 search converged on the combination *J1, Sunitinib and Bortezomib.*

Sr. No.	S.I. (kras)	S.I. (wt)	Therapeutic Index	Fit. ranks	Thr.Ind. -Std	Next Gen. candidate	No. drugs	Combinations
1	17	10.9	6.1	1	3.36	Yes	3	J1 - SUNITINIB - BORTEZOMIB
2	21.5	19.3	2.2	2		No	5	J1 - RAPAMYCIN - SUNITINIB - BORTEZOMIB - 5FU
3	9.6	9.7	-0.1	3		No	5	J1 - RAPAMYCIN - SUNITINIB - BORTEZOMIB - DOCETAXEL
4	13.4	14.2	-0.8	5		No	5	J1 - RAPAMYCIN - SUNITINIB - BORTEZOMIB - ERLOTINIB
5	2.1	3.6	-1.5	6		No	5	J1 - RAPAMYCIN - SUNITINIB - BORTEZOMIB - TRICHOSTATIN
6	14.8	15.3	-0.5	4		No	5	J1 - RAPAMYCIN - SUNITINIB - BORTEZOMIB - AAG 17
7	8.5	11.1	-2.6	8		No	4	J1 - RAPAMYCIN - SUNITINIB - BORTEZOMIB
8	8.8	11.3	-2.5	7		No	3	J1 - RAPAMYCIN - BORTEZOMIB
9	7.5	11.8	-4.3	10		No	5	J1 - RAPAMYCIN - SUNITINIB - BORTEZOMIB - DOXOROBICIN
10	1.6	5.8	-4.2	9		No	5	J1 - RAPAMYCIN - SUNITINIB - BORTEZOMIB - VIVOLUX 60
11	7.2	14.2	-7	11		No	5	J1 - RAPAMYCIN - SUNITINIB - CETUXIMAB - BORTEZOMIB
12	25	36.1	-11.1	12		No	3	RAPAMYCIN - SUNITINIB - BORTEZOMIB
13	15.3	29.8	-14.5	13		No	5	J1 - RAPAMYCIN - SUNITINIB - BORTEZOMIB - ACRIFLAVINIUM
14	19	38.1	-19.1	14		No	3	J1 - RAPAMYCIN - SUNITINIB

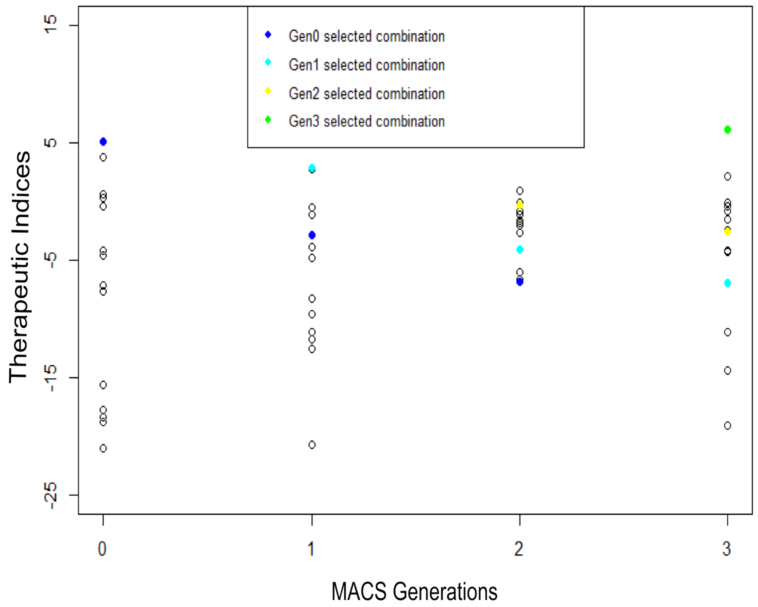

Figure S1: **Optimization overview of all iterations of pilot experiment:** Here the x-axis labeled "MACS generations" shows the consecutive algorithmic iterations and y-axis shows the therapeutic indices of combinations in an iteration. Selected/best combination in each iteration was assigned a color, the iteration 0 (random iteration) selection was assigned blue color, to track its position just follow the blue circles in different generations. Similarly, iteration 1 was assigned cyan, iteration 2 assigned yellow and iteration 3 assigned green color.

3 TACS algorithm implementation

Table S6: Results from generation 0 of TACS algorithm implementation. In this generation a combination of *rapamycin, 17AAG* is at the top that has TI 67. Another combination of *sunitinib,17AAG, Afungin* is the second best combination with TI 65.5. These two combinations were perturbed around to make the generation 1.

Sr. No.	S.I. (CCRF-CEM)	S.I. (HT29)	S.I. (HCT116)	Therapeutic Index	Fit. ranks	Thr.Ind. -Std	Next Gen. candidate	No. drugs	Combinations
1	95.9	84.3	78	15	9		No	2	Rapamycin - Sunitinib
2	18.5	24.2	4.6	4	11		No	6	Sunitinib - Mitomycin - Afungin - Rapamycin - Trichostatin A - 17AAG
3	33.4	39.8	19.5	4	10		No	4	Mitomycin - 17AAG - Rapamycin - Trichostatin A
4	63.6	50.5	43.5	17	8		No	4	Afungin - 17AAG - Mitomycin - Trichostatin A
5	85.1	72.5	47.8	25	6		No	4	Afungin - Sunitinib - Trichostatin A - Rapamycin
6	59	93.3	87.3	-31	12		No	2	Sunitinib - Mitomycin
7	97.3	40.4	28.5	63	3		No	4	Trichostatin A - Rapamycin - 17AAG - Afungin
8	55.6	87.2	86.8	-31	13		No	3	Rapamycin - Mitomycin - Afungin
9	106.2	86	67	30	4		No	2	Sunitinib - Trichostatin A
10	55.9	40.1	17.8	27	5		No	5	Mitomycin - Afungin - 17AAG - Sunitinib - Trichostatin A
11	111.4	47.6	41	67	1	65	Yes	2	Rapamycin - 17AAG
12	115.3	99.1	90	21	7		No	2	Trichostatin A - Afungin
13	95.9	84.3	78	65.5	2		Yes	3	Sunitinib - 17AAG - Afungin

Table S7: Results from generation 1 in TACS implementation experiment. Two top ranked combinations *sunitinib, 17AAG, afungin, trichostatin a* and *rapamycin, 17AAG, trichostatin a* were selected as parents of next generation.

Sr. No.	S.I. (CCRF-CEM)	S.I. (HT29)	S.I. (HCT116)	Therapeutic Index	Fit. ranks	Thr.Ind. -Std	Next Gen. candidate	No. drugs	Combinations
1	82.6	33.5	21.9	55	10		No	4	Sunitinib - 17AAG - Afungin - Rapamycin
2	54.4	30	22.5	28	11		No	4	Sunitinib - 17AAG - Afungin - Mitomycin
3	102.4	34	23	74	7		No	3	Sunitinib - 17AAG - Afungin
4	106.1	29.6	13.2	85	1	82.9	Yes	4	Sunitinib - 17AAG - Afungin - Trichostatin A
5	108.3	33.2	23.1	80	3		No	2	Sunitinib - 17AAG
6	97.4	67.5	89.4	19	13		No	2	Sunitinib - Afungin
7	114.6	31.4	45.3	76	6		No	2	17AAG - Afungin
8	91.6	31.2	23.1	64	9		No	3	Rapamycin - 17AAG - Sunitinib
9	54.9	28.7	37.3	22	12		No	3	Rapamycin - 17AAG - Mitomycin
10	105.9	23.3	23.1	83	2		Yes	3	Rapamycin - 17AAG - Trichostatin A
11	110.1	27	39.8	77	5		No	3	Rapamycin - 17AAG - Afungin
12	110.4	87.3	96.6	18	14		No	1	Rapamycin
13	110.4	26.9	36.3	79	4		No	2	Rapamycin - 17AAG
14	110.1	31.4	52.1	68	8		No	1	17AAG

Table S8: Results from generation 2 in TACS implementation experiment. A combination *sunitinib, 17AAG, afungin, trichostatin a* is the best combiantion in two consecutive genrations, hence triggered the stop of TACS algorithm.

Sr. No.	S.I. (CCRF-CEM)	S.I. (HT29)	S.I. (HCT116)	Therapeutic Index	Fit. ranks	Thr.Ind. -Std	Next Gen. candidate	No. drugs	Combinations
1	103.8	34.8	13.2	80	1	77.85		4	Sunitinib - 17AAG - Afungin - Trichostatin A
2	109.1	30.5	37.4	75	2			3	17AAG - Afungin - Trichostatin A
3	99.4	37.7	16.5	72	3			3	Sunitinib - 17AAG - Trichostatin A
4	97.9	28.5	26.7	70	5			3	Rapamycin - 17AAG - Trichostatin A
5	108	38	37.5	70	4			2	Rapamycin - 17AAG
6	101.4	41.1	24.9	68	7			3	Sunitinib - 17AAG - Afungin
7	96.7	31.2	25.4	68	8			4	Rapamycin - 17AAG - Trichostatin A - Afungin
8	87.6	31.5	6.4	69	6			5	Sunitinib - 17AAG - Afungin - Trichostatin A - Rapamycin
9	87.3	31.6	10	67	9			4	Rapamycin - 17AAG - Trichostatin A - Sunitinib
10	96	37.8	51.1	52	10			2	17AAG - Trichostatin A
11	94.7	77.8	74	19	12			3	Sunitinib - Afungin - Trichostatin A
12	38.4	28.1	9.7	20	11			5	Sunitinib - 17AAG - Afungin - Trichostatin A - Mitomycin
13	98.8	81.2	82.9	17	13			2	Rapamycin - Trichostatin A
14	32.1	27.6	22.1	7	14			4	Rapamycin - 17AAG - Trichostatin A - Mitomycin

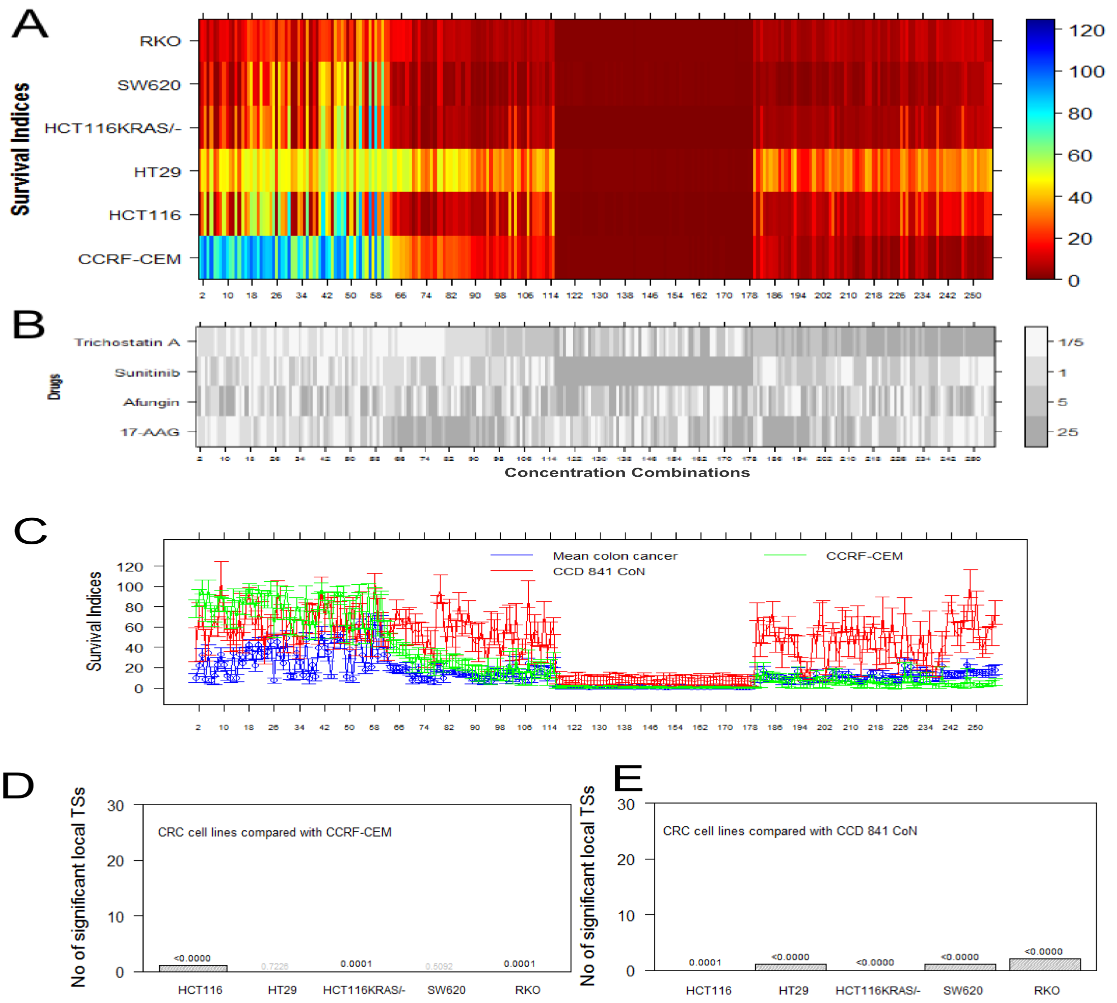

Figure S2: **Factorial concentration-response and TS (therapeutic synergy) study of combination (Sunitinib, 17-AAG, Afungin, Trichostatin A)**. Each of 256 different concentration combinations was tested against five CRC cell line models and two normal/reference/toxicity cell line models. The concentrations are color coded in panel B and were selected to be 1/5, 1, 5 and 25 times the IC_{20} concentration used in the combination search. **A**, Heatmap of SI values (%) for tested CRC cell lines, and the reference/toxicity model CCRF-CEM used in the iterative search, at the concentrations color coded in panel B. **B**, Heatmap of the 256 different concentrations tested, sorted by difference between SI of CCRF-CEM and mean SI of CRC cell lines. **C**, Graph of average SIs (concentrations are shown in B) across the five cancer cell lines as well as SI values for the two normal/reference/toxicity cell line models, CCRF-CEM and CCD 841 CoN. Error bars indicate 95% CI. **D**, Bar graph showing number of times it is found a concentration combination offers TS when performing 5 pairwise comparisons encompassing the 5 CRC cell lines versus the CCRF-CEM. For each comparison p value is provided that is an omnibus test for TS. **E**, Bar graph showing number of times it is found a concentration combination offers TS when performing 5 pairwise comparisons encompassing the 5 CRC cell lines versus the CCD 841 CoN. For each comparison p values is supplemented that is an omnibus test for TS.

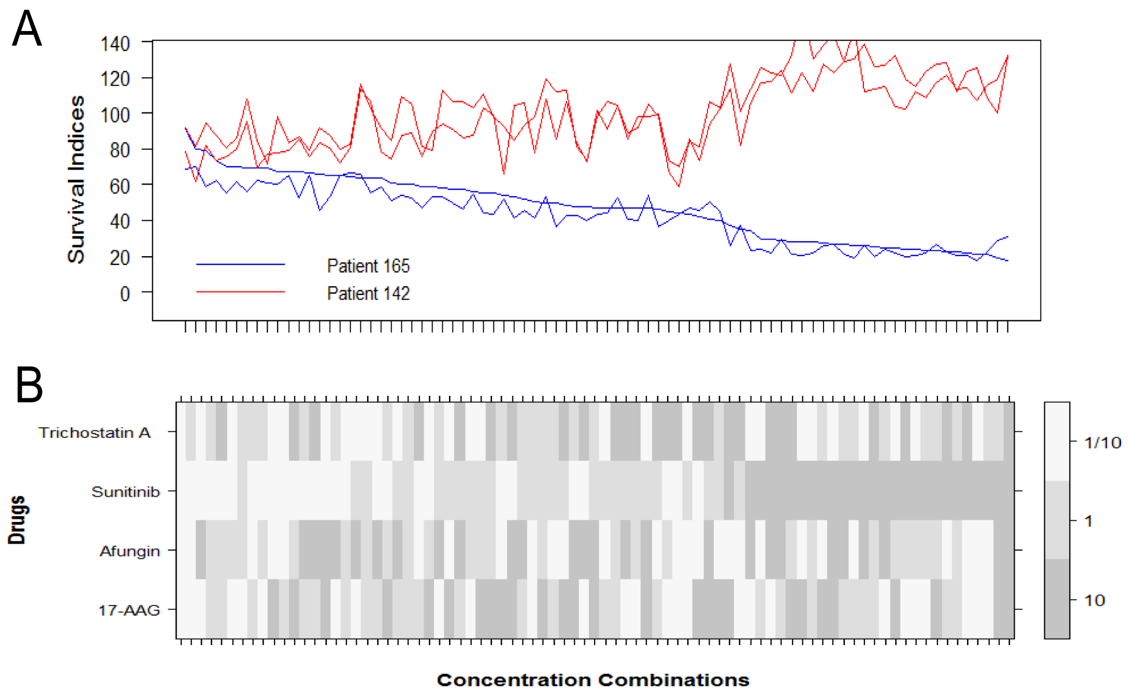

Figure S3: **Factorial concentration-response study of combination (Sunitinib, 17-AAG, Afungin, Trichostatin A) in patient cells performed at three different concentrations corresponding to 1/10, 1 and 10 times the concentration used in the interative search. A**, SI values for CRC patient cells tested twice. **B**, Concentrations corresponding to A expressed as fractions of the concentrations used the iterative search.

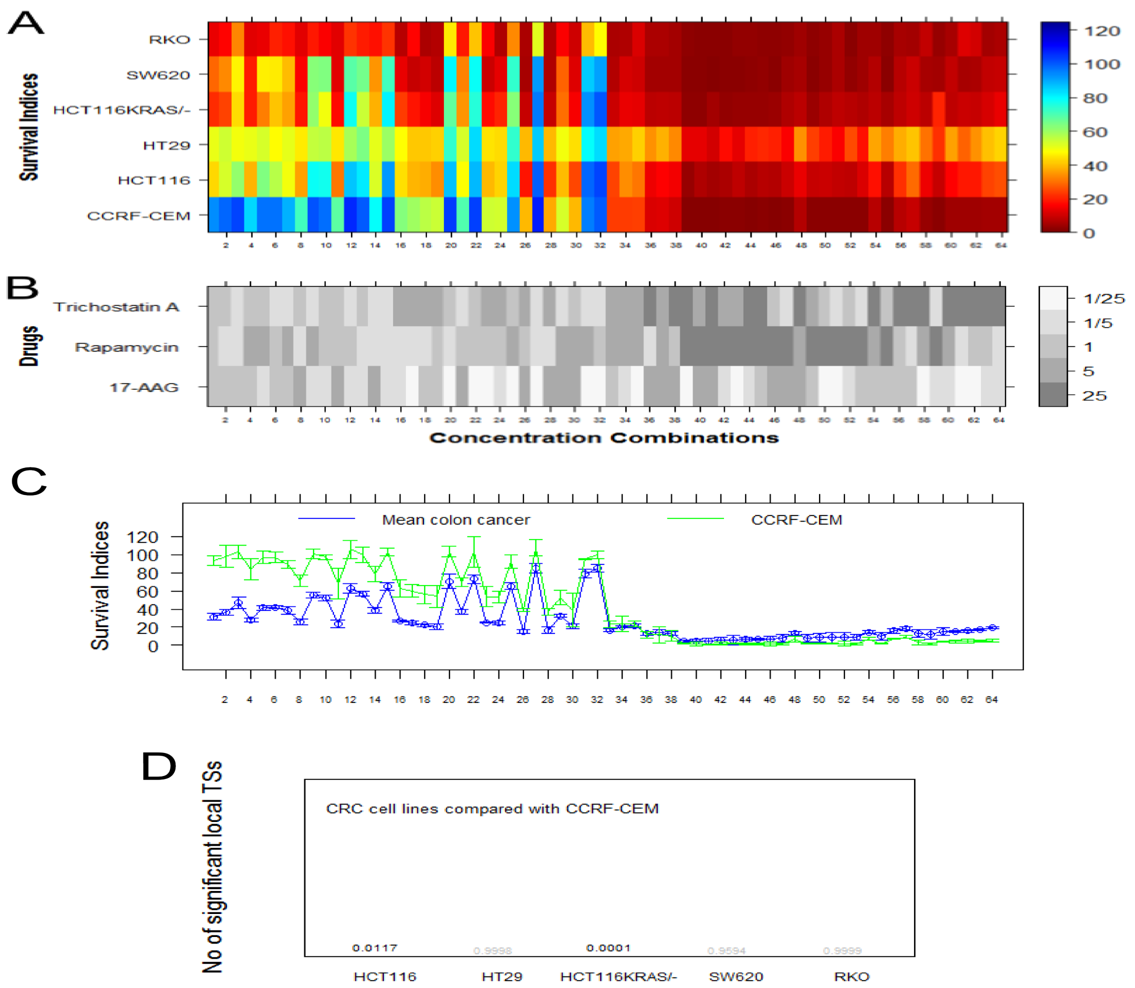

Figure S4: **Factorial concentration-response and TS study of combination (Rapamycin, 17-AAG, Trichostatin A)**. Each of 64 different concentration combinations was tested against five CRC cell line models and one normal/reference/toxicity cell line model. The concentrations are color coded in panel B and were selected to be 1/5, 1, 5 and 25 times the IC_{20} concentration used in the combination search for Trichostatin A and Rapamycin. For 17-AAG concentrations 1/25,1/5, 1 and 5 times of the IC_{20} used in iterative search were analyzed. **A**, Heatmap of SI values (%) for tested CRC cell lines, and the reference/toxicity model CCRF-CEM used in the iterative search, at the concentrations color coded in panel B. **B**, Heatmap of the 64 different concentrations tested, sorted by difference between SI of CCRF-CEM and mean SI of CRC cell lines. **C**, Graph of average SIs (concentrations are shown in B) across the five cancer cell lines as well as SI values for the one normal/reference/toxicity cell line model, CCRF-CEM. Error bars indicate 95% CI. **D**, Bar graph showing number of times it is found a concentration combination offers TS when performing 5 pairwise comparisons encompassing the 5 CRC cell lines versus the CCRF-CEM. For each comparison p value is provided that is an omnibus test for TS.

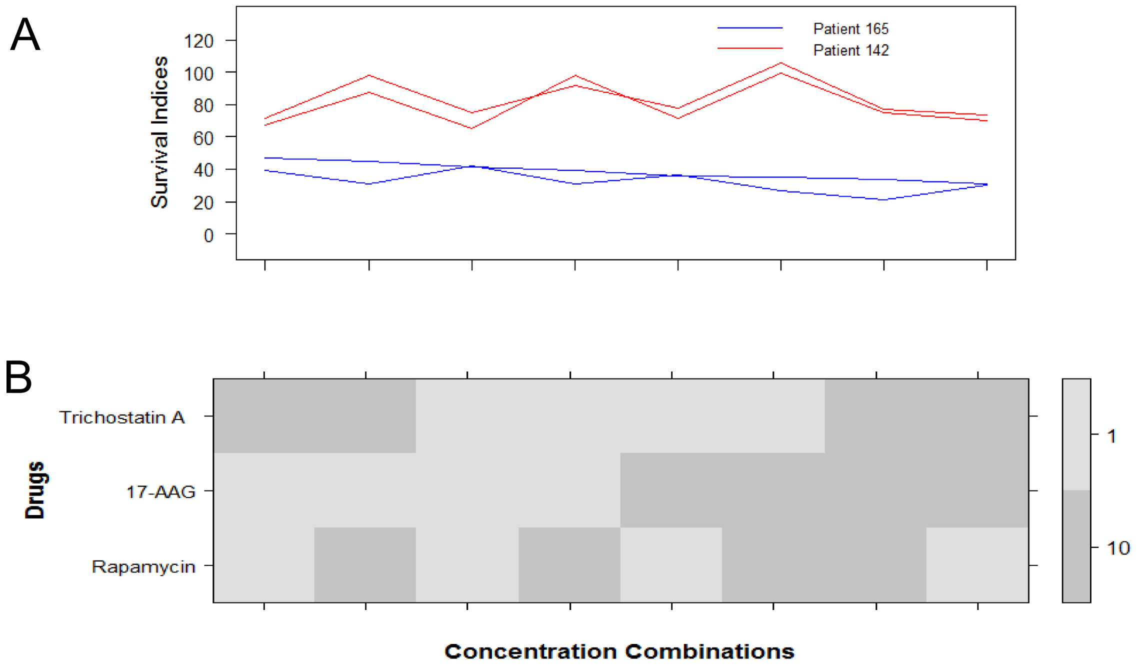

Figure S5: **Factorial concentration-response study of combination (Rapamycin, 17-AAG, Trichostatin A) in patient cells performed at two different concentrations corresponding to 1 and 10 times the concentration used in the interative search.** **A**, SI values for CRC patient cells tested twice. **B**, Concentrations corresponding to A expressed as fractions of the concentrations used the iterative search.

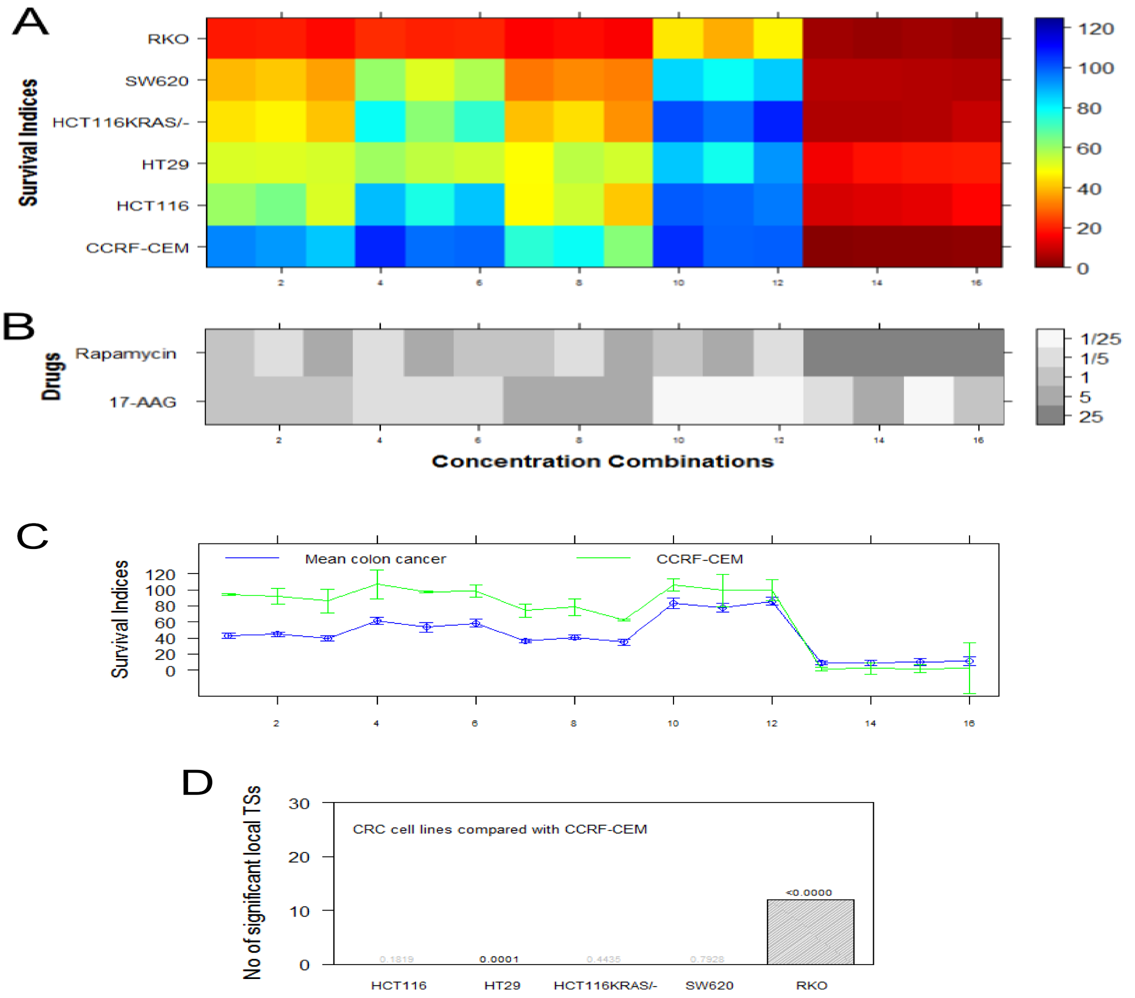

Figure S6: **Factorial concentration-response and TS study of combination (Rapamycin, 17-AAG).** Each of 16 different concentration combinations was tested against five CRC cell line models and one normal/reference/toxicity cell line model. The concentrations are color coded in panel B and were selected to be 1/5, 1, 5 and 25 times the IC_{20} concentration used in the combination search for Rapamycin. For 17-AAG concentrations 1/25,1/5, 1 and 5 times of the IC_{20} used in iterative search were analyzed. **A**, Heatmap of SI values (%) for tested CRC cell lines, and the reference/toxicity model CCRF-CEM used in the iterative search, at the concentrations color coded in panel B. **B**, Heatmap of the 16 different concentrations tested, sorted by difference between SI of CCRF-CEM and mean SI of CRC cell lines. **C**, Graph of average SIs (concentrations are shown in B) across the five cancer cell lines as well as SI values for the one normal/reference/toxicity cell line models, CCRF-CEM. Error bars indicate 95% CI. **D**, Bar graph showing number of times it is found a concentration combination offers TS when performing 5 pairwise comparisons encompassing the 5 CRC cell lines versus the CCRF-CEM. For each comparison p value is provided that is an omnibus test for TS.

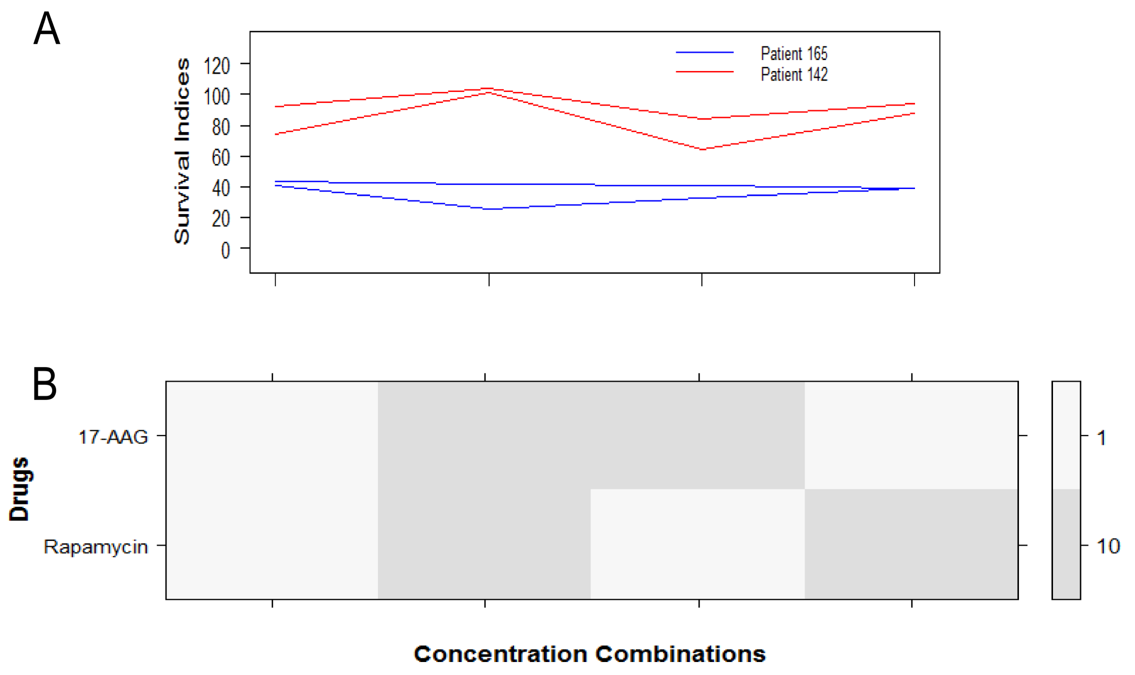

Figure S7: **Factorial concentration-response study of combination (Rapamycin, 17-AAG) in patient cells performed at two different concentrations corresponding to 1 and 10 times the concentration used in the interative search.** **A**, SI values for CRC patient cells tested twice. **B**, Concentrations corresponding to A expressed as fractions of the concentrations used the iterative search.

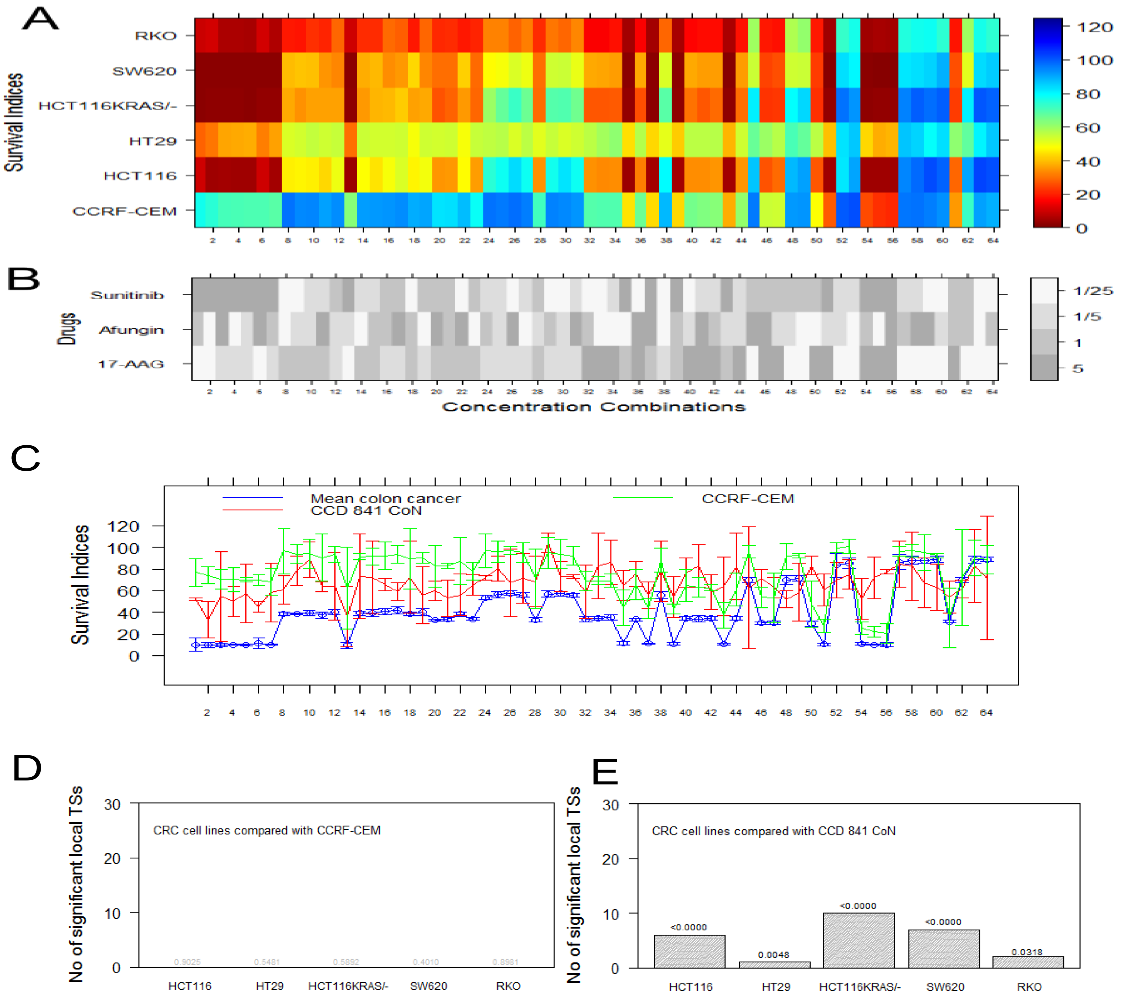

Figure S8: **Factorial concentration-response and TS study of combination (Sunitinib, 17-AAG, Afungin)**. Each of 64 different concentration combinations was tested against five CRC cell line models and two normal/reference/toxicity cell line models. The concentrations are color coded in panel B and were selected to be 1/25, 1/5, 1 and 5 times the IC_{20} concentration used in the combination search. **A**, Heatmap of SI values (%) for tested CRC cell lines, and the reference/toxicity model CCRF-CEM used in the iterative search, at the concentrations color coded in panel B. **B**, Heatmap of the 64 different concentrations tested, sorted by difference between SI of CCRF-CEM and mean SI of CRC cell lines. **C**, Graph of average SIs (concentrations are shown in B) across the five cancer cell lines as well as SI values for the two normal/reference/toxicity cell line models, CCRF-CEM and CCD 841 CoN. Error bars indicate 95% CI. **D**, Bar graph showing number of times it is found a concentration combination offers TS when performing 5 pairwise comparisons encompassing the 5 CRC cell lines versus the CCRF-CEM. For each comparison p value is provided that is an omnibus test for TS. **E**, Bar graph showing number of times it is found a concentration combination offers TS when performing 5 pairwise comparisons encompassing the 5 CRC cell lines versus the CCD 841 CoN. For each comparison p value is supplemented that is an omnibus test for TS.

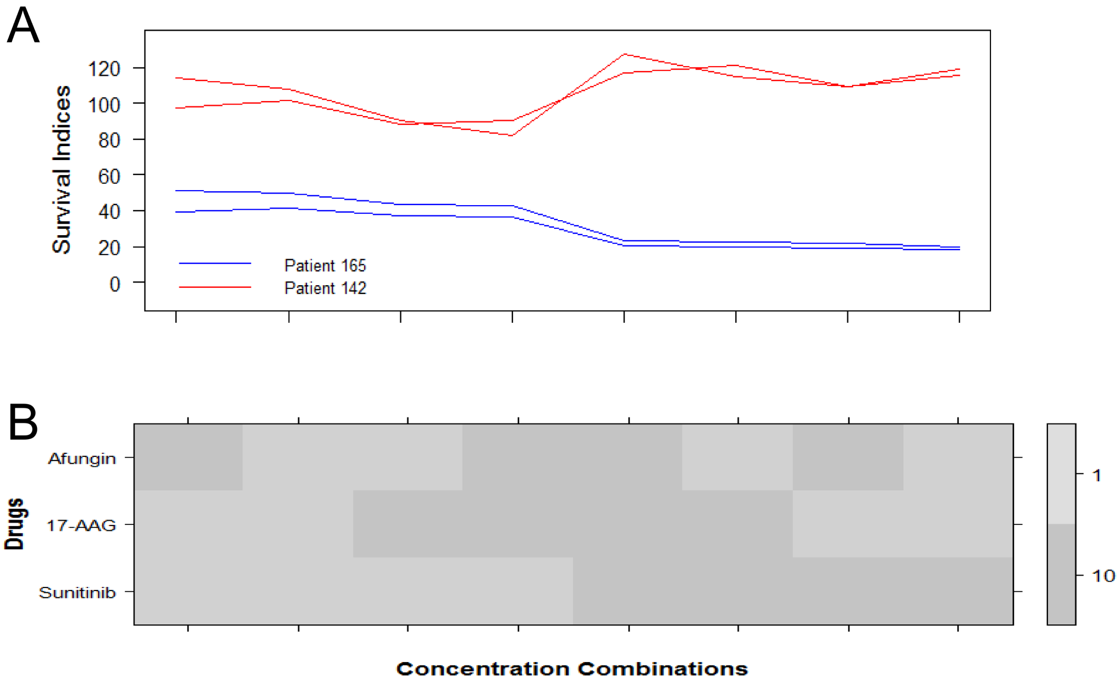

Figure S9: **Factorial concentration-response study of combination (Sunitinib, 17-AAG, Afungin) in patient cells performed at two different concentrations corresponding to 1 and 10 times the concentration used in the interative search.** **A**, SI values for CRC patient cells tested twice.**B**, Concentrations corresponding to A expressed as fractions of the concentrations used the iterative search.

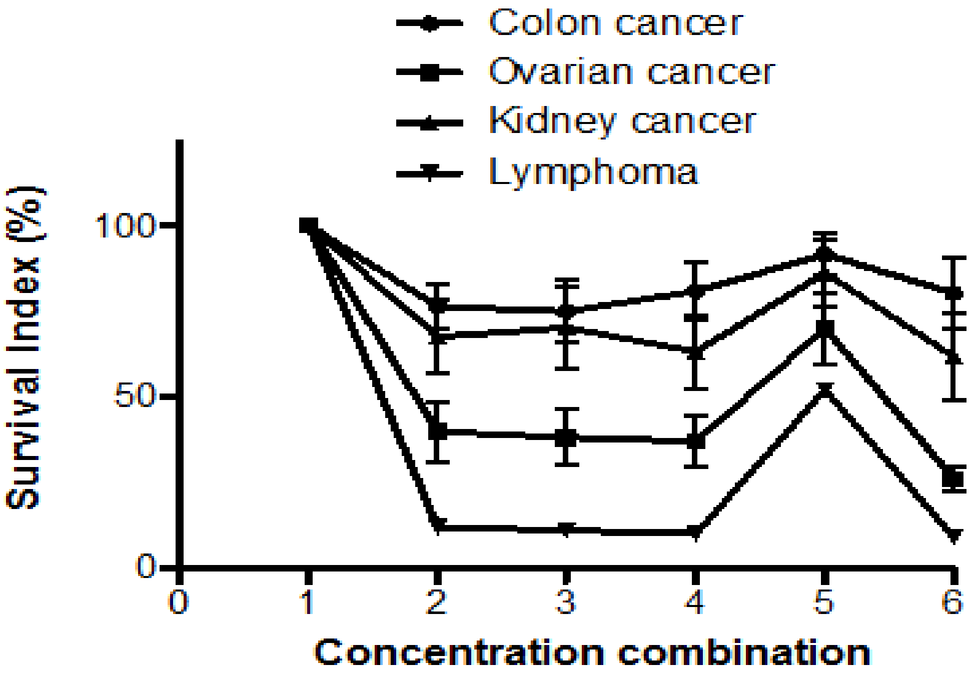

Figure S10: **Concentration-response study of combination (17-AAG, Afungin, Trichostatin A) in patient cells performed at five different concentrations.** Data are presented as mean survival index ±SE. Combination concentrations for 17-AAG/Afungin/ Trichostatin are as follows. **1:** unexposed control, **2:** 0.04/0.0004/0.4, **3:** 0.04/0.02/0.4, **4:** 0.04/0.05/0.4, **5:** 0.05/0.01/0.08, **6** 0.1/0.1/0.8. Numbers of samples were 11 for colorectal cancer, 9 for ovarian cancer, 6 for kidney cancer and 1 for lymphoma (no error bar).

References

[1] Zinner RG, Barrett BL, Popova E, Damien P, Volgin AY, Gelovani JG, et al. Algorithmic guided screening of drug combinations of arbitrary size for activity againt cancer cells. Mol Cancer Ther. 2009;8(3):521–532.

[2] Pinedo HM, Peters GF. Fluorouracil: biochemistry and pharmacology. JCO. 1988;6:1653–1664.

[3] Swift LP, Rephaeli A, Nudelman A, Phillips DR, Cutts MS. Doxorubicin-DNA Adducts Induce a Non-Topoisomerase II Mediated Form of Cell Death. Cancer Res. 2006;66:4863.

[4] Matsuo T, Nishizuka SS, Ishida A, Iwaya T, Ikeda M, Wakabayashi G. Analysis of the anti-tumor effect of cetuximab using protein kinetics and mouse xenograft models. BMC Research Notes. 2011;p. 4:140.

[5] Katherine LWA, C F. Docetaxel: A Review of its Use in Metastatic Breast Cancer. Drugs. 2005;65(17):2513–2531.

[6] Wickström M, Haglund C, Lindman H, Nygren P, Larsson R, Gullbo J. The novel alkylating prodrug J1: diagnosis directed activity profile ex vivo and combination analyses in vitro. Invest New Drugs. 2008;26(3):195–204.

[7] Tourneau CL, Raymond E, Faivre S. Sunitinib: a novel tyrosine kinase inhibitor. A brief review of its therapeutic potential in the treatment of renal carcinoma and gastrointestinal stromal tumors (GIST). Ther Clin Risk Manag. 2007;3(2):341–348.

[8] Cesare G, Anna BM, Clorinda S, Antonio R, Paolo M, Fortunato C. Erlotinib in Non-Small Cell Lung Cancer Treatment: Current Status and Future Development. The Oncologist. 2007;12:7840–849.

[9] Malti N, Thomas J, Hamsa P, Leigh C, Jerry A, Andrew KS. The proteasome inhibitor bortezomib sensitizes cells to killing by death receptor ligand TRAIL via BH3-only proteins Bik and Bim. Mol Cancer Ther. 2005;4:443.

[10] Sehgal SN. Sirolimus: its discovery, biological properties, and mechanism of action. Transplantation Proceedings. 2003;35 (Suppl 3A):7S–14S.

[11] Lee K, Zhang H, Qian DZ, Rey S, Liu JO, Semenza GL. Acriflavine inhibits HIF-1 dimerization, tumor growth and vascularization. Proc Natl Acad Sci. 2009;106(42):17910–17915.

[12] Kenno V, Christian AV, Frank PD, Josè MC, Dirk T, Paul G. Ab initio study of the binding of Trichostatin A (TSA) in the active site of Histone Deacetylase Like Protein (HDLP). Org Biomol Chem. 2003;1:2951–2957.

[13] Pelicano H, Carew JS, McQueen TJ, Andreeff M, Plunkett W, Keating MJ, et al. Targeting Hsp90 by 17-AAG in leukemia cells: mechanisms for synergistic and antagonistic drug combinations with arsenic trioxide and Ara-C. Leukemia. 2005;20:610–619.
